# An *ex vivo* test to investigate genetic factors conferring susceptibility to atypical haemolytic uremic syndrome

**DOI:** 10.3389/fimmu.2023.1112257

**Published:** 2023-02-09

**Authors:** Sara Gastoldi, Sistiana Aiello, Miriam Galbusera, Matteo Breno, Marta Alberti, Elena Bresin, Caterina Mele, Rossella Piras, Lucia Liguori, Donata Santarsiero, Ariela Benigni, Giuseppe Remuzzi, Marina Noris

**Affiliations:** Clinical Research Center for Rare Diseases Aldo e Cele Daccò and Centro Anna Maria Astori, Science and Technology Park Kilometro Rosso, Istituto di Ricerche Farmacologiche Mario Negri IRCCS, Bergamo, Italy

**Keywords:** rare variants, aHUS, complement, endothelial cells, endothelial C5b-9 formation

## Abstract

**Introduction:**

Comprehensive genetic analysis is essential to clinical care of patients with atypical haemolytic uremic syndrome (aHUS) to reinforce diagnosis, and to guide treatment. However, the characterization of complement gene variants remains challenging owing to the complexity of functional studies with mutant proteins. This study was designed: 1) To identify a tool for rapid functional determination of complement gene variants; 2) To uncover inherited complement dysregulation in aHUS patients who do not carry identified gene variants.

**Methods:**

To address the above goals, we employed an ex-vivo assay of serum-induced C5b-9 formation on ADP-activated endothelial cells in 223 subjects from 60 aHUS pedigrees (66 patients and 157 unaffected relatives).

**Results:**

Sera taken from all aHUS patients in remission induced more C5b-9 deposition than control sera, independently from the presence of complement gene abnormalities. To avoid the possible confounding effects of chronic complement dysregulation related to aHUS status, and considering the incomplete penetrance for all aHUS-associated genes, we used serum from unaffected relatives. In control studies, 92.7% of unaffected relatives with known pathogenic variants exhibited positive serum-induced C5b-9 formation test, documenting a high sensitivity of the assay to identify functional variants. The test was also specific, indeed it was negative in all non-carrier relatives and in relatives with variants non-segregating with aHUS. All but one variants in aHUS-associated genes predicted in-silico as likely pathogenic or of uncertain significance (VUS) or likely benign resulted as pathogenic in the C5b-9 assay. At variance, variants in putative candidate genes did not exhibit a functional effect, with the exception of a *CFHR5* variant. The C5b-9 assay in relatives was helpful in defining the relative functional effect of rare variants in 6 pedigrees in which the proband carried more than one genetic abnormality. Finally, for 12 patients without identified rare variants, the C5b-9 test in parents unmasked a genetic liability inherited from an unaffected parent.

**Discussion:**

In conclusion, the serum-induced C5b-9 formation test in unaffected relatives of aHUS patients may be a tool for rapid functional evaluation of rare complement gene variants. When combined with exome sequencing the assay might be of help in variant selection, to identify new aHUS-associated genetic factors.

## Introduction

Atypical haemolytic uremic syndrome (aHUS) is a rare but usually severe form of HUS which in the past resulted in end-stage kidney disease (ESKD) in up to two thirds of affected patients (1).

Mutations in genes that encode complement factor H (FH), factor I (FI), and factor B (FB), membrane-cofactor protein (MCP), C3 and thrombomodulin (THBD), and anti-FH antibodies have been found in 50-60% of patients with primary aHUS, documenting the crucial role of uncontrolled complement activation in the pathogenesis of the disease (2). Penetrance is incomplete for all aHUS-associated genes as documented by finding within pedigrees of several unaffected carriers (2).

The introduction of anti-complement therapy has dramatically improved the outcome of aHUS patients (3, 4). Eculizumab is a humanised anti-C5 antibody that blocks C5 cleavage into C5a and C5b. The latter participates in the assembly of the terminal C5b-9 membrane attack complex (MAC). Eculizumab inhibits the common final step of all complement pathways, while preserving upstream complement functions, such as opsonisation.

Ever since therapeutic complement inhibition was approved, speedy recognition of complement-dependent aHUS has become a major diagnostic issue. However, at present, the differential diagnosis of aHUS from other thrombotic microangiopathies (TMA) is essentially made after ruling out severe ADAMTS13 deficiency (thrombotic thrombocytopenic purpura, TTP), infection with Shiga-like toxin producing bacteria (STEC-HUS), or coexisting conditions assumed to be the etiologic disease factor (secondary HUS) (5). In addition, the duration and optimal dose of treatment are often debated, particularly given the extremely high costs of the drug (6). Results from a prospective multicentre study of eculizumab discontinuation in adult and paediatric aHUS patients (7) showed that carriers of a rare complement gene variant had an increased risk of relapse, and proposed that complement genetics could help selecting patients who will undergo eculizumab discontinuation.

Thus, a comprehensive genetic analysis is essential to clinical care for aHUS patients in order to confirm the diagnosis and guide treatment (8, 9). However, genetic testing still takes too long and fails to identify an underlying complement genetic abnormality in 40% of patients. In addition, the definition of the identified variants in complement genes as pathogenic or benign remains challenging and time-consuming, given the complexity of functional studies with recombinant or plasma purified mutants (9). As a result, a high number of variants are defined as variants of uncertain significance (VUS). The interpretation of aHUS-associated gene variants is further complicated by the fact that penetrance is largely incomplete; indeed, in over 90% of cases the variant is inherited from an unaffected parent and may be present in other unaffected relatives.

This study was designed based on two objectives: 1) To identify a tool for rapid functional determination of rare complement gene variants; 2) To find genetic forms of aHUS among patients without an identified gene variant in known disease-associated genes.

In order to address the above goals, we used a published ex vivo assay based on ADP-activated confluent human microvascular endothelial cells (HMEC-1) that are exposed to serum from carriers of aHUS-associated complement gene variants (10). This assay reproduces the 2-hit model of aHUS, according to which one or more gene abnormalities predispose to aHUS, but a trigger that perturbs endothelial cells is required for terminal complement activation on cell surface, and aHUS to occur (10).

We hypothesised that if a complement gene variant causes a genetic liability to complement dysregulation, this can be unmasked by measuring C5b-9 formation on endothelium incubated with serum from unaffected relatives who carry the gene variant. The latter will provide the advantage versus their proband of not having confounding complement-activating factors that are associated with the chronic aHUS status in their circulation. Carriers of variants with pathogenic significance will exhibit higher-than-normal serum-induced C5b-9 formation, whereas carriers of variants with no functional impact will have a negative test result.

## Methods

### Study participants

Participants were patients with primary aHUS and their available relatives, recruited from those included in the International Registry of Recurrent and Familial Haemolytic Uremic Syndrome/Thrombotic Thrombocytopenic Purpura (HUS/TTP) between 2008 and 2021. The Registry was established in 1996 at the Aldo e Cele Daccò Clinical Research Center for Rare Diseases (Ranica, Bergamo) (villacamozzi.marionegri.it/seu).

**Figure 1:**
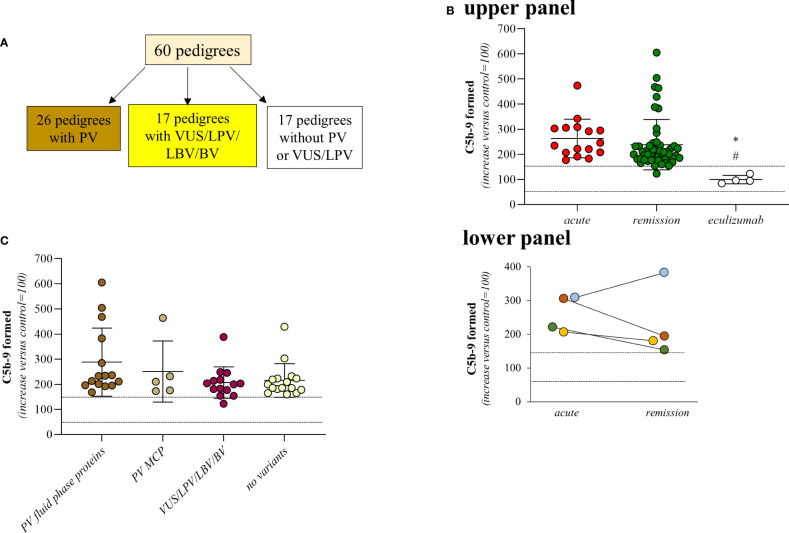
Serum from aHUS patients induces higher-than-normal C5b-9 formation on activated HMEC-1. **(A)** Distribution of rare complement gene variants among the 60 pedigrees. **(B)** Upper Panel, Endothelial surface area covered by C5b-9 staining after 2h incubation of ADP-activated HMEC-1 with serum (diluted 1:2 in test medium) from aHUS patients. Serum from aHUS patients was collected during the acute phase of the disease (n=16), or during remission (n=50), or during eculizumab treatment (n=4). Dotted lines are upper and lower limits of normal range. **Lower Panel,** Endothelial surface area covered by C5b-9 staining after 2h incubation of ADP-activated HMEC-1 with serum (diluted 1:2 in test medium) from aHUS patients. Serum from aHUS patients (n=4) was collected both during the acute phase of the disease and during the remission. Dotted lines are upper and lower limits of normal range. **(C)** Endothelial surface area covered by C5b-9 staining after 2h incubation of ADP-activated HMEC-1 with serum (diluted 1:2 in test medium) from aHUS patients who were in remission. aHUS patients were subdivided into carriers of pathogenetic variants (PV) in genes encoding fluid phase complement proteins (n=15), or pathogenetic variants (PV) in the *MCP* gene only (n=5), or VUS/LPV/LBV/BV (n=14) or non-carrier of rare variants (n=16). Dotted lines are upper and lower limits of normal range. The results are shown as the fold increase of stained surface area after incubation with aHUS serum versus control pool of sera run in parallel. Points represent fold increase values of single subjects. Horizontal bars are mean ± SD values. *P<0.01 vs aHUS patients in acute phase, #P<0.05 vs aHUS in remission. PV, pathogenic variant; LPV, likely pathogenic variant; VUS, variant of uncertain significance; LBV/BV, likely benign/benign variant.

For this retrospective study we analysed all available samples from pedigrees with primary aHUS (n=60) for whom genetic data and serum samples were available, both from the probands and from the relatives. Sixty-six patients who met the inclusion criteria (probands, n=60; affected relatives, n=6) and 157 healthy relatives for a total of 223 subjects were studied. Among the probands, 14 have a familial history of the disease (**Figure 1A** and **Supplementary Table 1**).

Patients were studied at different time points: 1) during the acute phase before any treatment (n=16); 2) while in remission, without treatment for >6 months (n=50); 3) in remission under standard eculizumab treatment for the patients (n=4) for whom only samples taken during eculizumab treatment were available. Four patients were studied both during the acute phase and while in remission (**Supplementary Table 1**). The samples used for this study were stored at Centro Risorse Biologiche (CRB) “Mario Negri”, Biobank “Malattie Rare e Malattie Renali”.

Diagnosis of HUS was made in patients who have had one or more episodes of microangiopathic haemolytic anaemia and thrombocytopenia, with haematocrit (Ht) <30%, haemoglobin (Hb) <10 g/dL, serum lactate dehydrogenase (LDH) >500 IU/L, undetectable haptoglobin, fragmented erythrocytes in the peripheral blood smear, and platelet count <150,000/μL, associated with acute renal failure (serum creatinine >1.3 mg/dL for adults, >0.5 mg/dL for children below 5 years of age and >0.8 mg/dL for children aged 5-10, and/or urinary protein/creatinine ratio >200 mg/g; or an increase in serum creatinine or the urinary protein/creatinine ratio >15% compared to baseline levels) (11). TTP was ruled out on the basis of ADAMTS13 activity >10% and no anti-ADAMTS13 antibodies.

Primary aHUS was diagnosed in HUS patients after ruling out both secondary underlying conditions and infections with Stx-*E.Coli.*

Stx-*E.Coli* infection was defined as the presence of *stx* and *eae* genes (by PCR) or Shiga-toxin (Vero cell assay) in the stool and/or antibodies anti Shiga-toxin (ELISA) and/or LPS O157, O26, O111, or O145 (ELISA) in serum. For patients, the Stx-*E.Coli* tests were processed centrally by the Food Safety, Nutrition and Veterinary Public Health Department, Istituto Superiore di Sanità, Rome, Italy.

Secondary aHUS was diagnosed in patients with underlying malignant hypertension, autoimmune diseases, other glomerulopathies, haematopoietic stem cell or solid organ transplantation, and cancer.

Full remission was defined as the normalisation of both haematological parameters (Ht >30% and Hb >10 g/dL and LDH <500 IU/L and platelets >150,000/μL) and renal function (serum creatinine <1.3 mg/dL for adults, <0.5 mg/dL for children below 5 years of age and <0.8 mg/dL for children aged 5-10; urinary protein/creatinine ratio <200 mg/g). Haematological remission in patients was defined as the normalisation of haematological parameters but with renal dysfunction.

The protocol was approved by the Ethical Committee of the Azienda Sanitaria Locale Bergamo, Italy. Participants or their legal guardians provided written informed consent.

### Complement genetic abnormalities and anti-CFH autoantibodies

The screening of coding sequences of aHUS-associated genes (*CFH, CFHR1, MCP, CFI, CFB, C3, THBD* and *DGKE*) and candidate genes (*CFHR2, CFHR3, CFHR4, CFHR5*, and *C5*) was performed using amplicon-based next-generation sequencing (12). Rare functional variants (missense, nonsense, indel, or splicing variants) with minor allele frequency (MAF) <0.01 in Genome Aggregation Database (gnomAD, https://gnomad.broadinstitute.org/) were selected. Stop-gain, frameshift and splicing variants, and missense variants with published functional studies, were categorized as pathogenic variants (PV). The other variants were categorized as likely pathogenic (LPV), variants of uncertain significance (VUS), likely benign or benign, using guidelines from the American College of Medical Genetics and Genomics (ACMG) and from the KDIGO conference on aHUS and C3G (5, 13, 14). We also used the Combined Annotation Dependent depletion (CADD) version 1.6, which is a tool for scoring the deleteriousness of single nucleotide variants as well as insertion/deletion variants, which integrates multiple annotations into one metric (https://cadd.gs.washington.edu/info) (14, 15). We selected the PHRED-scaled C-score ranking; variants with CADD scores >10 were defined “potentially pathogenetic”. The search for genomic abnormalities that affect *CFH* and *CFHR* genes (16, 17) was undertaken using multiplex ligation-dependent probe amplification as reported (18). We also genotyped the *CFH* single-nucleotide polymorphisms (SNPs) (c.1-331C>T, rs3753394; c.184G>A, p.V62I, rs800292; c.1204C>T, p.H402Y, rs1061170; c.2016A>G, p.Q672Q, rs3753396; c.2237-543G>A, rs1410996; c.2808G>T, p.E936D, rs1065489) that define the disease risk haplotype *CFH*_TGTGGT_ (known as *CFH*–H3 haplotype) and one SNP in *MCP* (rs7144, c.∗897T>C) that tags the risk *MCP*_GGAAC_ haplotype (11, 19–21). Anti-FH autoantibodies were measured in plasma using ELISA (22).

### Serum-induced C5b-9 deposition on HMEC-1

The assay was performed as described previously (6, 10), with minor modifications. The human microvascular endothelial cells (HMEC-1 cell line) were a gift from Dr Edwin Ades and Francisco J. Candal of CDC and Dr Thomas Lawley of Emory University, Atlanta, GA. As growth medium we used MCDB 131 (Gibco, Grand Island, NY) supplemented with 10% foetal bovine serum (Gibco), 10 µg/mL hydrocortisone, 100 U/mL penicillin, 100 µg/mL streptomycin, 2 mM glutamine (Gibco), and 50 µg/mL endothelial cell growth factor. For each experiment the procedure was standardized by plating the same number of cells (75.000) that were cultured for 96 hours and left in serum-free medium for 24 hours. Thereafter the integrity and confluence of the cell monolayer was checked at phase-contrast microscope. If a slide did not full-fill the criteria, it was discarded. Cells were washed 3 times with test medium (HBSS: 137 mM NaCl, 5.4 mM KCl, 0.7 mM Na_2_HPO_4_, 0.73 mM KH_2_PO_4_, 1.9 mM CaCl_2_, 0.8 mM MgSO_4_, 28 mM Trizma base pH 7.3, 0.1% dextrose; with 0.5% BSA) and activated with 10 µM ADP (Sigma) for 10 minutes. Thereafter cells were exposed for 2 hours to serum from patients or healthy relatives or healthy controls, 50% in test medium (HBSS with 0.5% BSA). HMEC-1 were then fixed in 3% paraformaldehyde, blocked with PBS with 2% BSA and stained with rabbit anti-human complement C5b-9 complex antibody (Calbiochem, catalog number 204903, 1h at room temperature, 1/200 final dilution) followed by FITC-conjugated secondary antibody (Jackson Immuno Research Laboratories, catalog number 111-095-144, 1h at room temperature, 1/50 final dilution). We have previously validated the rabbit anti-human C5b-9 antibody using the mouse monoclonal anti-human C9 antibody (W13-15, Hycult Biotech, 1h at room temperature, 1/50 final dilution) that recognizes human C9 neoantigen. Results of C5b-9 formation on ADP-activated HMEC-1 exposed to aHUS serum with the rabbit anti-human C5b-9 were comparable to those with the anti-human C9 antibody (23).

Sera from 10 different healthy blood donors were pooled and the pool was run in each experiment as a reference (100%) for C5b-9 staining. We also analysed single sera from additional controls (n=35), tested separately as independent samples. The 35 donors did not overlap with the 10 donors of the pool. For each of them we calculated the percentages of C5b-9 deposits vs. control serum pool to set the normal range (mean ± 2SD of the percentage of C5b-9 deposits of the single control sera vs. control serum pool: ADP-activated HMEC-1, 60-149%) (**Supplementary Figure 1**).

In addition, in each experiment, we added the serum from an aHUS patient that had shown a strong positivity on ADP-activated HMEC-1 in previous experiments, as positive control. As negative control, in random experiments we added sCR1 (an inhibitor of the classical, alternative and lectin pathway of complement activation, 150 µg/ml, R&D System) to the tested serum (**Supplementary Figure 2**).

We verified the integrity of the cell monolayer after exposure to serum samples in parallel slides in which cells were stained with May-Grunwald Giemsa.

The fluorescent staining on the endothelial cell surface was acquired using the Apotome Axio Imager Z2 (Zeiss) microscope. Fifteen fields, which were systematically digitised along the surface were acquired using a computer-based image analysis system. The nuclear staining with DAPI (4’,6-diamidino-2-phenylindole, Sigma-Aldrich) was used as guide to determine if a fluorescent area was on HMEC-1 surface. The operator discarded the fields where cell confluence was missing. The exposure time was constant. The area occupied by the fluorescent staining was evaluated with automatic edge detection using the built-in specific functions of the software Image J (NIH, Bethesda, MD), and calculated as pixel^2^ per field analysed (10). For each sample, 15 fields were analysed; the highest and the lowest values were discarded and the mean of pixel^2^ the other 13 fields was calculated. We expressed the results as the percentage of the staining compared to the pixel^2^ of the control serum pool (used as denominator), run in parallel (10). All the images were acquired, and the staining was calculated by the same operator (S.G.), who during the analysis was blinded about the nature of the samples. The results were confirmed by a second blinded operator (M.G.).

We previously documented (10) the good reproducibility of the assay at the concordance correlation test, by testing twice in different experiments the samples from several patients (10). As further check of good reproducibility of the assay, in the present study we performed biological replicates by analysing samples collected at 2 different time points in remission from 6 aHUS patients. As shown in **Supplementary Figure 3**, elevated C5b-9 formation on activated endothelial cells was confirmed in biological replicates.

### Serum C3 and plasma sC5b-9 evaluation

For patients from which enough samples were available, we evaluated serum C3 and plasma sC5b-9 levels. Complement C3 was measured in serum by nephelometry. Normal ranges (defined as mean ± 2SD) for C3: 83-180 mg/dL, n=50. SC5b-9 levels were evaluated in EDTA plasma by MicroVue sC5b-9 Plus EIA (SC5b-9 Plus; Quidel). Normal range of plasma sC5b-9 in our laboratory: 127-400 ng/mL; n=50.

### Statistical analysis

Comparisons of groups were performed with one-way analysis of variance (ANOVA).

Sensitivity of serum-induced C5b-9 formation (positive test: staining in pixel^2^ significantly higher in the test sample than in the control sample, run in parallel) was calculated as: number of test samples with positive test/samples with positive results + false negatives.

The GraphPad Prism 8.3.0 software was used for statistical analysis unless otherwise stated. P values of less than 0.05 were considered to be statistically significant.

## Results

### Serum from aHUS patients caused higher-than-normal C5b-9 formation on activated endothelial cells

A total of 66 patients with primary aHUS from 60 pedigrees (60 probands and 6 affected relatives **Figure 1A**) were analysed with the serum-induced C5b-9 formation test on ADP-activated HMEC-1. Consistent with previously published data (6, 10), sera from all patients with acute aHUS studied before therapy (plasma or eculizumab) (n=16) and sera from all but one patient studied while in remission without therapy for >6 months (n=50), induced higher-than-normal (263 ± 77%, and 238 ± 101%, respectively, >149% of normal human serum pool) C5b-9 formation on ADP-activated HMEC-1 (**Figure 1B**, upper panel). As shown in **Figure 1B** lower panel, results obtained with serum from patients studied both during acute phase and in remission (n=4) showed similarly elevated C5b-9 formation on activated endothelial cells.

In agreement with our previous work (10), only a fraction of patients showed circulating markers of complement activation, such as reduced serum C3 and increased plasma sC5b-9, both during the acute phase (reduced C3: 10/16; increased sC5b-9: 3/6) or during remission (reduced C3: 20/47; increased sC5b-9: 6/14) (**Supplementary Table 1**). These results are in line with the cell surface restricted complement activation that characterizes aHUS. Of note, no correlations were found between C3 serum levels and C5b-9 formation on HMEC-1 (Spearman’s coefficient of rank correlation: P=0.2095), and between sC5b-9 levels and C5b-9 formation on HMEC-1 (Spearman’s coefficient of rank correlation: P=0.2750).

C5b-9 formation on endothelial cells exposed to sera from the four aHUS patients on eculizumab treatment was in the control range (99 ± 17%) (**Figure 1B**, upper panel).

We then divided the 50 patients who were in remission and out of treatment, on the basis of their genetic background. We did not observe any significant difference in serum-induced C5b-9 formation among the following subgroups: 1) patients with known rare pathogenic variants (PV) that affect fluid phase proteins (288 ± 136%, n=15); 2) patients with only PV that affect the cell surface protein MCP (251 ± 122%, n=5); 3) patients with variants of uncertain significance/likely pathogenic/likely benign/benign variants (VUS/LPV/LBV/BV) (207 ± 63%, n=14); 4) patients without identified genetic or acquired complement abnormalities (215 ± 67%, n=16) (**Figure 1C**; **Tables 1**, **2A**). None of the studied patients had anti-FH antibodies.

**Table 1:** List of known rare pathogenic variants (PV) identified in aHUS patients.

family code	gene	nucleotide change	aminoacid change	variant effect	gnomAD frequency	classification	Dam/Pred	CADD (versione1.3)	references (Doi)
#020, #856	*CFH*	c.3628C>T	p.R1210C	missense	0.0001451	pathogenic	1/11	11.77	10.1086/344515
#101	*CFH*	c.232A>G	p.R78G	missense	–	pathogenic	6/11	15.77	10.1074/jbc.M110.211839
#267, #120, #265	*CFH*	c.3572C>T	p.S1191L	missense	–	pathogenic	4/11	18.67	10.1002/humu.9408
#45	*CFH*	c.3547T>A	p.W1183R	missense	–	pathogenic	–	23.9	10.1086/344515
#251	*CFH*	c.3572C>T + c.3590T>C	p.S1191L + p.V1197A	*CFH/CFHR*1 gene conversion	- 0.0000071	pathogenic	4/11- 7/11	18.67-19.52	10.1002/humu.9408
#52, #210	*CFH*	c.3590T>C	p.V1197A	missense	0.0000071	pathogenic	7/11	19.52	10.1002/humu.9408
#603, #81	*CFH*	c.3514G>T	p.E1172X	stop gained	0.000012	pathogenic	–	39	10.1172/JCI16651
#390	*CFH*	c.3493+1G>A	–	splice donor	0.000004	pathogenic	2/2	23.6	10.1016/j.kint.2017.07.015
#192	*CFI*	c.1446_1450delTTCAC	p.L484Vfs3X	frameshift	0.0000279	pathogenic	–	27.9	10.1016/j.molimm.2007.05.004
#563, #158	*C3*	c.1774C>T	p.R592W	missense	0.000004	pathogenic	7/12	34	10.4049/jimmunol.1701695; 10.1182/blood-2008-01-133702
#380, #551	*CFHR1/CFH hybrid*	reverse hybrid *CFHR1 1-5 – CFH 23*	–	hybrid	–	pathogenic	–	–	10.1681/ASN.2013121339
#1245	*CFHR1/CFH hybrid*	reverse hybrid *CFHR1 1-4 – CFH 22-23*	–	hybrid	–	pathogenic	–	–	10.1681/ASN.2017050518
#316	*CFHR1*	c.869T>C + c.887C>T	p.L290S + p.A296V	*CFHR1/CFH* gene conversion	0.0000264 – 0.000034	pathogenic	0/11	0.002 – 0.392	10.1681/ASN.2017050518
#176	*CFH/CFHR1 hybrid*	hybrid *CFH 1-21 – CFHR1 5-6*	–	hybrid	–	pathogenic	–	–	10.1002/humu.9408
#1065 #646, #782	*MCP*	c.286+2T>G	–	splice donor	0.0000521	pathogenic	2/2	14.54	10.1016/j.molimm.2006.07.004
#458	*MCP*	c.760delT + c.770_772delATA	p.F254Lfs43X	frameshift	–	pathogenic	–	27.6	–
#192	*MCP*	c.287-2A>G	–	splice acceptor	0.0000251	pathogenic	2/2	20.1	10.1182/blood-2005-10-007252
#2816	*MCP*	c.282dupT	p.Y95Lfs2X	frameshift	–	pathogenic	0/0	23.3	–

**Table 2A:** List of rare likely pathogenetic variants (LPV), variants of uncertain significance (VUS) and benign and likely benign variants in genes encoding soluble proteins and identified in aHUS patients.

family code	gene	nucleotide change	aminoacid change	variant effect	gnomAD frequency	Dam/Pred n*	CADD	classification	references (Doi)
**#2880^**	***CFH***	**c.2957-7A>G**	**-**	**intronic/splice region**	**0.0002**	**0/0**	**0.267**	**likely benign**	**-**
**#155**	***CFH***	**c.2908A>G**	**p.I970V**	**missense**	**0.00000796**	**1/3**	**0.010**	**VUS°**	**10.1182/blood-2005-10-007252; 10.4049/jimmunol.1701695**
**#194**	***CFH***	**c.3544_3545insGGTGGACAGCCAAACAGAAGCTTTATTCGAGAACAG**	**p.R1182delinsRWTAKQKLYSRTG**	**insertion**	**-**	**0/0**	**-**	**LPV**	**-**
**#2813**	***CFH***	**c.127C>A**	**p.P43T**	**missense**	**0.00000398**	**3/11**	**20.9**	**VUS**	**-**
**#390**	***CFH***	**c.1548T>A**	**p.N516K**	**missense**	**0.0003049**	**8/11**	**16.62**	**VUS°**	**10.4049/jimmunol.1701695; 10.1111/j.1600-6143.2008.02297.x; 10.1182/blood.2021012037**
**#181**	***CFH***	**c.2686_2700delAAATTGAGTTATACT**	**p.896_900delKLSYT**	**deletion**	**-**	**-**	**16.8**	**LPV°**	**10.4049/jimmunol.1701695**
**#52**	***CFH***	**c.2932T>C**	**p.W978R**	**missense**	**-**	**11/11**	**25.6**	**LPV**	**-**
**#736**	***CFH***	**c.2171C>A**	**p.T724K**	**missense**	**0.00009201**	**6/11**	**24.1**	**VUS**	**-**
#265	*CFI*	c.1661A>T	p.E554V	missense	0.00001415	4/4	25.4	VUS°	10.1681/ASN.2012090884; 10.2215/CJN.02210310; 10.1093/hmg/ddv091; 10.4049/jimmunol.1701695
#646	*CFI*	c.1322A>G	p.K441R	missense	0.003575	2/12	0.002	likely benign°	10.4049/jimmunol.1701695
**#593**	***CFI***	**c.560G>A**	**p.R187Q**	**missense**	**0.00007780**	**2/12**	**17.91**	**VUS°**	**10.4049/jimmunol.1701695**
#380	*C3*	c.2284G>A	p.V762I	missense	0.00001193	0/12	0.254	VUS	10.2215/CJN.02210310
**#2169**	***C3***	**c.181G>A**	**p.D61N**	**missense**	**0.00001988**	**6/12**	**19.03**	**LPV**	**-**
**#2880^**	***C3***	**c.962G>T**	**p.G321V**	**missense**	**-**	**8/12**	**26.3**	**LPV**	**-**
**#1485^**	***C3***	**c.74+1delG**	**-**	**intronic/splice region**	**-**	**0/0**	**23.6**	**VUS**	**-**
**#1077**	***C3***	**c.1898A>G**	**p.K633R**	**missense**	**0.0004565**	**1/12**	**0.043**	**VUS°**	**10.4049/jimmunol.1701695; 10.1182/blood-2009-05-221549**
**#4**	***C3***	**c.485C>G**	**p.T162R**	**missense**	**-**	**4/12**	**14.34**	**VUS°**	**10.4049/jimmunol.1701695; 10.2215/CJN.02210310**
#856	*CFB*	c.1778+8C>T	-	intronic/splice region	0.00005677	0/0	7.153	VUS°	10.4049/jimmunol.1701695
**#1485^**	***CFB***	**c.1018A>T**	**p.N340Y**	**missense**	**-**	**6/12**	**13.56**	**VUS**	**-**
**#267, #623**	***C5***	**c.1060C>A**	**p.L354M**	**missense**	**0.005561**	**6/11**	**25**	**benign+**	**-**
**#2071**	***C5***	**c.4924T>G**	**p.L1642V**	**missense**	**0.00006765**	**0/12**	**14.44**	**VUS+**	**-**
#1683	*C5*	c.3029C>T	p.A1010V	missense	0.0007	5/12	23.9	VUS	-
**#412^**	***CFHR2***	**c.325A>G**	**p.T109A**	**missense**	**0.0058**	**3/11**	**14.5**	**benign**	**-**
**#20**	***CFHR4***	**c.758delG**	**p.C253Lfs15X**	**frameshift**	**-**	**-**	**25.0**	**VUS**	**-**
**#1279**	***CFHR4***	**CFHR1-CFHR4 duplication**	**-**	**-**	**-**	**-**	**-**	**VUS**	**10.1159/000497823**
#101	*CFHR4*	c.799+3A>C	-	intronic/splice region	0.00098	0/0	2.578	likely benign	-
**#1494**	***CFHR4***	**c.1058T>A**	**p.I106N**	**-**	**-**	**-**	**12.97**	**VUS**	**-**
#563	*CFHR4*	c.1207A>T	p.N156Y	missense	-	-	22.7	VUS	-
#267	*CFHR5*	c.1412G>A	p.G471E	missense	0.0006083	6/11	22.3	VUS	10.4049/jimmunol.1701695
**#412^**	***CFHR5***	**c.485_486dupAA**	**p.E163Kfs10X**	**frameshift**	**0.0063**	**0/0**	**22.7**	**LPV°**	**-**

° databases of complement gene mutation (https://www.complement-db.org/home.php).

* Dam/Pre n: number of damaging predictions/total number of predictions. Variants carried by at least one unaffected relative are in bold characters.

^ Pedigrees with combined variants.

+: ClinVar; https://www.ncbi.nlm.nih.gov/clinvar.

**Table 2B:** List of rare complement gene variants identified in unaffected aHUS relatives but not in probands.

family code	gene	nucleotidechange	aminoacidchange	varianteffect	gnomAD allele frequency	CADD	*references (Doi)*
#623	*CFI*	c.1044+9G>A	–	intronic/spliceregion	0.00001989	2.153	-
#1650	*CFI*	c.1642G>C	p.E548Q	missense	0.0008207	29.1	10.4049/jimmumol.1701695°
#155	*CFB*	c.1037-10C>G	-	intronicregion	0.00073	7.632	-
#603	*CFB*	c.1270+6T>C	-	intronicregion/spliceregion	-	15.8	-
#623	*C5*	c.130G>T	p.Y44D	missense	-	24.4	-
#265	*CFHR2*	c.212C>T	p.T71M	missense	0.00398	18.04	
#1998	*CFHR4*	c.643C>G	p.P215A	missense	-	12.83	-

° database of complement gene mutation (https://www.complement-db.org/home.php).

Four out of 5 patients in remission with only *MCP* PVs also had one or two risk haplotypes in circulating complement factors, such as *CFH* (H3) and or CFHR1*B (**Supplementary Table 1**), which could contribute to complement dysregulation (20, 24).

Altogether, the above findings show that the test for C5b-9 formation on ADP-activated HMEC-1 with aHUS serum cannot provide enough information about the impact (if any) of the underlying genetic abnormalities on complement dysregulation on the cell surface.

### Serum from unaffected relative carriers of complement pathogenic variants induces C5b-9 formation on activated endothelial cells

**Figure 2:**
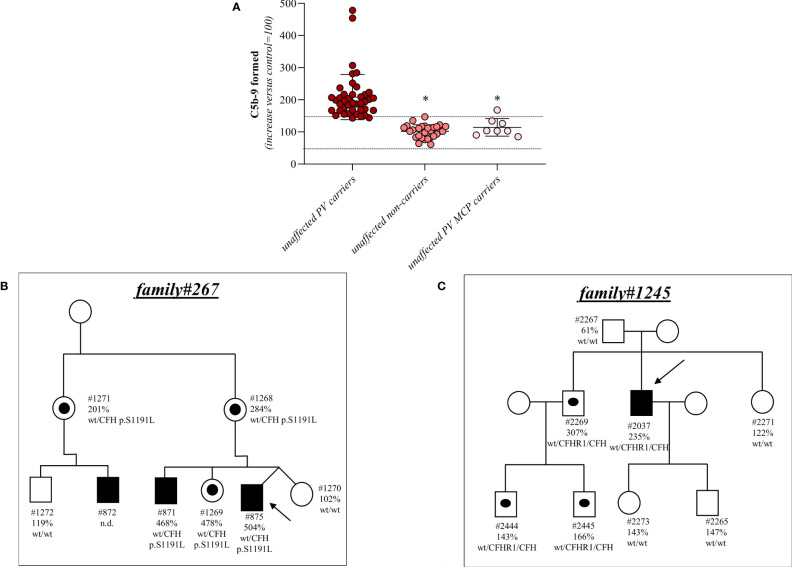
Serum from unaffected relative carriers of PV induces higher than normal C5b-9 formation on HMEC-1. **(A)** Endothelial surface area covered by C5b-9 staining after 2h incubation of ADP-activated HMEC-1 with serum (diluted 1:2 in test medium) from either unaffected carriers of PV in genes encoding for fluid phase complement proteins (n=41), or from unaffected non-carriers of PV (n=28) or from unaffected carriers of PV in the *MCP* gene only (n=8). The results are shown as the fold increase of stained surface area after incubation with serum from unaffected carriers versus control pool of sera run in parallel. Points represent fold increase values of single subjects. Horizontal bars are mean ± SD values. Dotted lines are upper and lower limits of normal range. *P<0.0001 vs. unaffected carriers of PV. **(B)** Pedigree #267 of aHUS proband #875 (indicated by the arrow) with a PV in CFH p.S1191L. The same PV was found in his brother (affected, #871), his mother (#1268), his sister (#1269), his uncle (#1271) and his cousin (affected, #872). **(C)** Pedigree #1245 of aHUS proband #2037 (indicated by the arrow) with a pathogenic *CFHR1/CFH* hybrid gene (*CFHR1_1-4_-CFH_22-23_*). The same hybrid was also found in his brother (#2269) and his nephews (#2444 and #2445). In each pedigree, the affected subjects are in black, healthy carriers of PV are identified with a black dot. The circles indicate females and the squares indicate males.

Among the 26 pedigrees with PV, we identified 14 PV in genes that encode circulating complement proteins or regulators (*CFH*, n=7; *CFI*, n=1; *C3*, n=1; *CFHR1/CFH* gene conversion, n=1; hybrid genes n=3, *CFH/CFHR1* gene conversion n=1, **Tables 1**, **3**) (25, 26). To evaluate the sensitivity of the ex vivo endothelial test to identify subjects carrying functional complement gene variants, we analysed serum from 41 healthy relatives who carried (from here on, unaffected relative carriers) the above PV, used as positive controls. Thirty-eight out of 41 carriers exhibited a higher-than-normal range (>149% of normal human serum pool) of serum-induced C5b-9 formation on ADP-activated HMEC-1 (208 ± 70%, n=41; **Figure 2A**) resulting in 92.7% sensitivity to identify carriers of a PV. In the same pedigrees, we were able to analyse 28 relatives who do not carry the above PV, who were used as negative controls. Serum from all of these subjects induced normal C5b-9 formation on endothelial cells (healthy relatives non-carrier of PV, 102 ± 20%, n=28; **Figure 2A**). We repeated the assay with serum from a subgroup of unaffected carriers (PV, n=3) or non-carriers (n=7) on unstimulated HMEC-1, and results showed normal C5b-9 formation with all sera (**Supplementary Figure 4**), confirming that the ex vivo assay can reproduce the “two hit” model of aHUS.

**Table 3:** Results of C5b-9 test in unaffected family members who carry PV in genes encoding fluid phase proteins that segregate with the disease.

gene	nucleotide change	aminoacid change	C5b-9 formed *(% increase versus control=100)*	n° carriers positive C5b-9 test *(>149%)*
*CFH*	c.3628C>T	p.R1210C	171%	6/7
			281%	
			144%	
			175%	
			454%	
			204%	
			186%	
*CFH*	c.232A>G	p.R78G	199%	2/2
			240%	
*CFH*	c.3572C>T	p.S1191L	284%	10/10
			478%	
			201%	
			155%	
			168%	
			151%	
			168%	
			187%	
			167%	
			196%	
*CFH*	reverse hybrid *CFHR1 _1-5_ – CFH_2_*_3_	Hybrid	208%	2/2
			216%	
*CFH*	hybrid *CFH _1-21_ – CFH_5-6_*	Hybrid	184%	1/1
*CFH*	reverse hybrid *CFHR1 _1-4_ – CFH_22-23_*	Hybrid	307%	2/3
			166%	
			143%	
*CFH*	c.3547T>C	p.W1183R	157%	1/1
*CFH*	c.3514G>T	p.E1172X	209%	2/2
			252%	
*CFH*	c.3590T>C	p.V1197A	205%	3/3
			167%	
			217%	
*CFH*	c.3493+1G>A	–	194%	1/1
*CFH*	*CFH/CFHR1* gene conversion c.3572C>T + c.3590T/C*	p.S1191L + p.V1197A	216%	1/1
*CFI*	c.1446_1450delTTCAC	p.L484Vfs3X	190%	1/1
*C3*	c.1774C>T	p.R592W	155%	5/6
			148%	
			237%	
			151%	
			223%	
			207%	
*CFHR1*	*CFHR1/CFH* gene conversion c.869T>C + c.887C>T	p.L290S + p.A296V	189%	1/1
Overall				38/41

* doi: 10.1002/humu.9408.

Results for 2 representative pedigrees are shown in **Figure 2B** (#267) and **Figure 2C** (#1245). The detailed clinical and family history of the 2 pedigrees are reported in the **Supplementary Material**.

In contrast, all but one unaffected relative carriers of PV only in the surface complement regulator MCP exhibited C5b-9 formation on ADP-activated HMEC-1 in the normal range (114 ± 27%, n=8 from 6 pedigrees) (**Figure 2A** and **Table 4**). Notably, the only unaffected MCP carrier showing higher than normal serum-induced C5b-9 formation also had the H3 haplotype, indicating a possible contribution of this haplotype in complement dysregulation, as we observed in affected MCP carriers.

**Table 4:** Results of C5b-9 test in unaffected family members who carry PV in MCP segregating with the disease.

gene	nucleotide change	aminoacid change	C5b-9 formed *(% increase versus control=100)*	n° carriers positive C5b-9 test *(>149%)*
*MCP*	c.286+2T>G	-	85%	0/5
			90%	
			104%	
			103%	
			103%	
*MCP*	c.760delT	-	134%	0/1
*MCP*	c.282dupT	p.Y95Lfs2X	168%	1/1
*MCP*	c.287-2A>G	-	126%	0/1
Overall				1/8

### Serum-induced C5b-9 formation test in unaffected carriers helps with the functional characterisation of gene variants

Based on the results of the serum-induced C5b-9 formation test that we found with the carriers of PV, we used the same approach to characterise rare variants predicted to be LPV or VUS, or likely benign or benign in silico. In the probands from 28 pedigrees, we identified 30 rare variants – classified as LPV, VUS, likely benign, or benign – in aHUS-associated genes that encode soluble proteins (*CFH, CFI, C3, CFB* and *CFHR1*, **Table 2A**) or in candidate complement genes (*C5, CFHR2, CFHR4* and *CFHR5*, **Table 2A**). For 22 variants we found at least one unaffected carrier relative, for a total of 27 relatives.

**Figure 3:**
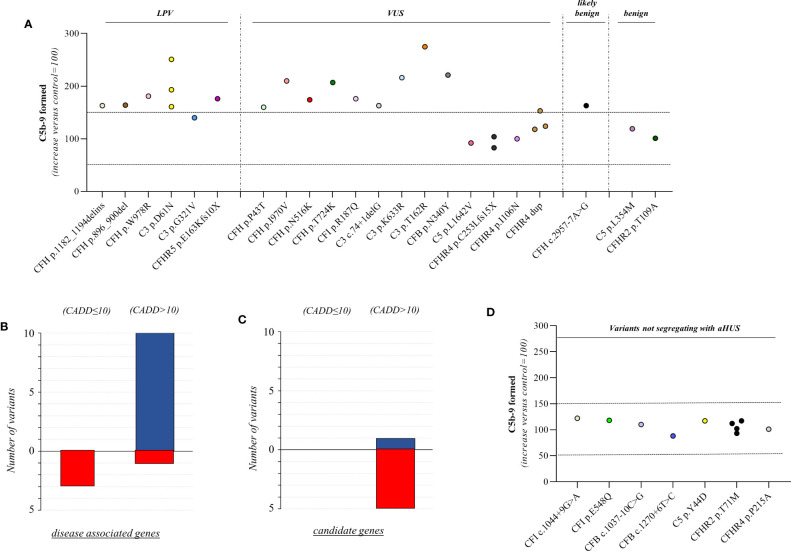
C5b-9 formation on HMEC-1 exposed to serum from unaffected relative carriers of rare complement gene variants. **(A)** Endothelial surface area covered by C5b-9 staining after 2h incubation of ADP-activated HMEC-1 with serum (diluted 1:2 in test medium) from carriers of variants likely pathogenic (LPV, n=8), of uncertain significance (VUS, n=16), likely benign (n=1), or benign (n=2). Results are shown as fold increase of stained surface area after incubation with serum from unaffected carriers versus control pool of sera run in parallel. Points represent fold increase values for single subjects. Dotted lines are upper and lower limits of normal range. **(B)** Breakdown of CADD computational predictions using a default score value cutoff >10 in disease-associated genes. (CADD ≤ 10: benign prediction; CADD > 10: pathogenic prediction) **(C)** Breakdown of CADD computational predictions using a default score value cutoff >10 in candidate genes. Red columns indicate variants with incorrect prediction; Blue columns indicate variants with correct predictions. **(D)** Endothelial surface area covered by C5b-9 staining after 2h incubation of ADP-activated HMEC-1 with serum (diluted 1:2 in test medium) from carriers of rare variants that do not segregate with aHUS (n=10). Results are shown as fold increase of stained surface area after incubation with serum from carriers of rare variants not segregating with aHUS versus control pool of sera run in parallel. Points represent fold increase values of single subjects. Dotted lines are upper and lower limits of normal range.

We found excessive C5b-9 formation on ADP-activated HMEC-1 in 18 of 27 relatives, indicating that they may have a genetic liability to complement dysregulation on the cell surface (**Figure 3A**; **Table 5**).

**Table 5:** Results of C5b-9 formation test in family members who carry VUS/LPV/LBV or benign variants.

gene	nucleotide change	aminoacid change	classification	gnomAD frequency	CADD	C5b-9 formed *(% increase vs control=100)*	n° carriers positive C5b-9 test *(>149%)*
							
*CFH*	c.127C>A	p.P43T	VUS	0.000003983	20.9	160%	1/1
*CFH*	c.2957-7A>G	-	likely benign	0.0002	0.267	163%	1/1
*CFH*	c.2908A>G	p.I970V	VUS	0.000007955	0.010	210%	1/1
*CFH*	c.3544_3545insGGTGGACAGCCAAACAGAAGCTTTATTCGAGAACAG	p.R1182delinsRWTAKQKLYSRTG	LPV	-	0.006	163%	1/1
*CFH*	c.1548T>A	p.N516K	VUS	0.0003049	16.62	174%	1/1
*CFH*	c.2686_2700delAAATTGAGTTATACT	p.896_900delKLSYT	LPV	-	16.80	164%	1/1
*CFH*	c.2932T>C	p.W978R	LPV	-	25.6	181%	1/1
*CFH*	c.2171C>A	p.T724K	VUS	0.00009201	24.1	207%	1/1
*CFI*	c.560G>A	p.R187Q	VUS	0.00007780	17.91	176%	1/1
*C3*	c.181G>A	p.D61N	LPV	0.00001988	19.03	161%	3/3
						251%	
						193%	
*C3*	c.962C>T	p.G321V	LPV	-	26.3	140%	0/1
*C3*	c.74+1delG	-	VUS	-	23.6	163%	1/1
*C3*	c.1898A>G	p.K633R	VUS	0.0004565	0.043	216%	1/1
*C3*	c.485C>G	p.T162R	VUS	-	14.34	275%	1/1
*CFB*	c.1018A>T	p.N340Y	VUS	-	13.56	221%	1/1
*C5*	c.4924C>G	p.L1642V	VUS	0.00006765	14.44	92%	0/1
*C5*	c.1060A>C	p.L354M	benign	0.005561	25	119%	0/1
*CFHR2*	c.325A>G	p.T109A	benign	0.0058	14.5	101%	0/1
*CFHR4*	c.758delG	p.C253Lfs15X	VUS	-	25	104%	0/2
						83%	
*CFHR4*	c.1058T>A	p.I106N	VUS	-	12.97	100%	0/1
*CFHR4*	*CFHR1-CFHR4* dup	-	VUS	-	-	153%	1/3
						133%	
						124%	
*CFHR5*	c.485_486dupAA	p.E163Kfs10X	LPV	0.0063	22.7	176%	1/1
Overall							18/27

Specifically, the test was positive in 7 out of 8 (87.5%), 10 out of 16 (62.5%), 1 out of 1 and 0 out of 2 subjects with LPV, VUS, likely benign or benign variants, respectively. Notably, among subjects with VUS, all the 6 that exhibited a negative C5b-9 test had variants only in putative candidate genes (*C5* and *CFHR4*). As observed with carriers of PV, when the assay with sera from unaffected carriers of LPV/VUS was repeated on unstimulated HMEC-1 (n=7), results showed normal C5b-9 formation for all subjects (**Supplementary Figure 4**).

Finally, in a few pedigrees with carriers of LPV (n=3) or VUS (n=4) that showed a positive test we could analyse sera from at least one non-carrier relative (**Supplementary Table 2**), all of which formed normal amount of C5b-9 on activated HMEC-1. To summarise, the results showed that all but one variants in aHUS-associated genes that were predicted in silico to be LPV, or VUS as well as a likely benign variant, were experimentally shown to be pathogenic. At variance, variants in putative candidate genes did not exhibit a functional effect in the C5b-9 test, with the only exception of a LPV in the *CFHR5* gene that showed a positive test (**Figure 3A**).

We further predicted variant pathogenicity computationally using CADD, adjusting the CADD score threshold for pathogenicity to >10. As shown in **Figure 3B**, 10 of 14 variants in disease-associated genes exhibited concordance between CADD and the results of the C5b-9 test, in contrast with only 1 of 6 variants in putative candidate genes (**Figure 3C**).

Among the 60 studied pedigrees we identified 7 rare variants that did not segregate with the disease (**Table 2B**), since they were present in unaffected relatives (n=10) but not in the probands. The C5b-9 test was negative in all of these subjects (**Figure 3D**), independently of their classification and CADD values (**Table 6**), supporting the potential of the test to differentiate between variants that do or do not have functional consequences on complement activation on the cell surface.

**Table 6:** Results of C5b-9 formation test in family members who carry variants not segregating with the disease.

gene	nucleotide change	aminoacid change	gnomAD allele frequency	CADD	C5b-9 formed *(% increase versus control=100)*	n° carriers positive C5b-9 test *(>149%)*
*CFI*	c.1044+9G>A	-	0.00001989	2.153	122%	0/1
*CFI*	c.1642G>C	p.E548Q	0.0008207	29.1	118%	0/1
*CFB*	c.1037-10C>G	-	0.00073	7.632	110%	0/1
*CFB*	c.1270+6T>C	-	-	15.8	88%	0/1
*C5*	c.130G>T	p.Y44D	-	24.4	117%	0/1
*CFHR2*	c.212C>T	p.T71M	0.00398	18.04	112%	0/4
					102%	
					117%	
					93%	
*CFHR4*	c.643C>G	p.P215A	-	12.83	101%	0/1
Overall						0/10

**Figure 4:**
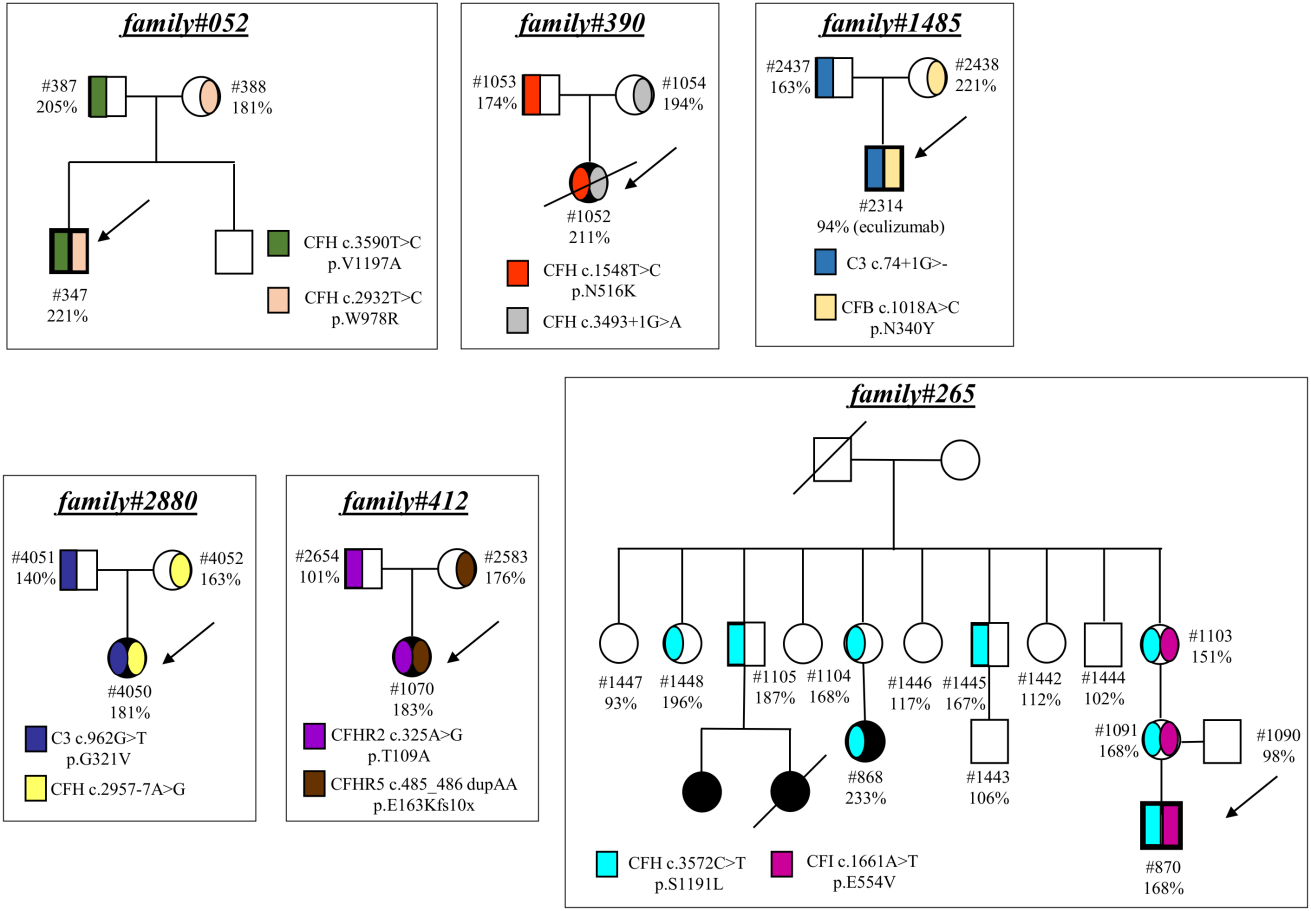
Pedigrees with combined variants. Pedigree #052 of the proband #347. Genetic analysis revealed a pathogenic variant in *CFH* (p.V1197A) inherited from the father (#387, green) and also a LPV in *CFH* (p.W978R) inherited from the mother (#388, pink). Pedigree #390 of the aHUS proband #1052. Genetic analysis revealed a pathogenic variant in *CFH* (c.3493+1G>A) inherited from the mother (#1054, grey) and a rare VUS in *CFH* (p.N516K) inherited from the father (#1053, red). Pedigree #265 of proband #870 carrying a pathogenic variant in *CFH* (p.S1191L, light blue) inherited from the mother and also present in the maternal affected relative (#868). Genetic analysis in the proband also found a VUS in *CFI* (p.E554V, purple) inherited from the mother but absent in the maternal affected relative. Pedigree #1485 of proband #2314. Genetic analysis revealed a VUS in *C3* (c.74+1delG) inherited from the father (#2437, blue) and a VUS in *CFB* (p.N340Y) inherited from the mother (#2438, orange). Arrows indicate probands, affected subjects are in black. Pedigree #2880 of the proband #4050. Genetic analysis revealed a LPV in *C3* (p.G321V) inherited from the father (#4051, deep blue) and a likely benign variant in *CFH* (c.2957-7A>G) inherited from the mother (#4052, yellow). Pedigree #412 of the proband #1070. Genetic analysis revealed a benign variant in *CFHR2* (p.T109A) inherited from the father (#2654, purple) and a LPV in *CFHR5* (p.E163Kfs10x) inherited from the mother (#2583, brown). Arrows indicate probands, affected subjects are in black. The circles are used for female subjects and squares for male subjects.

Finally, in 6 pedigrees the probands exhibited combined PV/LPV (family #52, **Figure 4**), or PV/VUS (family #390, and family #265, **Figure 4**), or combined VUS (family #1485, **Figure 4**), or combined LPV/likely benign variants (family #2880, **Figure 4**) or combined LPV/benign variants (family #412, **Figure 4**). The test was positive in unaffected carriers of the PV *CFH* p.V1197A and *CFH* c.3493+1G>A (**Figure 4**). The results of the C5b-9 test in unaffected carrier relatives indicated a functional pathogenic role for the *CFH* p.W978R LPV (CADD 25.6), the *CFH* p.N516K VUS (CADD 16.62), the *C3* c.74+1delG>- VUS (CADD 23.6), the *CFB* p.N340Y VUS (CADD 13.6), the *CFH* c.2957-7A>G likely benign (CADD 26.3) and the *CFHR5* p.E163Kfs10x LPV (CADD 22.7) (**Figure 4**). As for the *CFI* p.E554V VUS (CADD 25.4) in pedigree #265, the test was non-informative because we did not find an unaffected relative who carried this variant alone. However, it is likely non-functional since it did not segregate with the disease in this family because it was present in only one of the two affected subjects (**Figure 4**). The detailed clinical and family histories of the 6 pedigrees are reported in the **Supplementary Material**.

### Serum-induced C5b-9 formation test in trios of patients without variants in known genes may identify new genetic forms of aHUS

In 17 pedigrees we did not identify rare variants in disease-associated or candidate complement genes in the probands. To evaluate whether any of the above probands had inherited, from the unaffected parent, an unknown genetic abnormality predisposing to complement activation on the endothelial surface, we analysed C5b-9 formation in the probands and their parents (trios).

**Figure 5:**
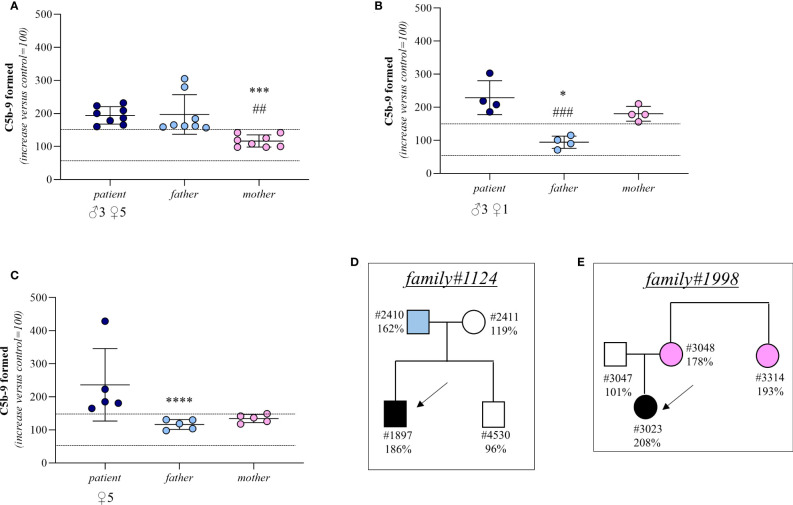
C5b-9 formation on activated HMEC-1 induced by serum from aHUS patients without identified mutations and from their parents. **(A–C)** Endothelial surface area covered by C5b-9 staining after 2h incubation of ADP-activated HMEC-1 with serum (diluted 1:2 in test medium) from aHUS patients without identified mutations (remission, n=16; acute, n=1) and their parents. Dotted lines are upper and lower limits of normal range. Results are shown as fold increase of stained surface area after incubation with serum from aHUS patients and their parents versus control pool of sera run in parallel. Points represent fold increase values of single subjects. Horizontal bars are mean ± SD values. *P<0.001, ***P<0.01, ****P<0.05 vs patient; ##P<0.01, ###P<0.05 vs other parent. **(D)** Pedigree #1124 of patient #1897 without identified mutations. **(E)** Pedigree #1998 of patient #3023 without identified mutations. Arrows indicate probands, affected individuals are in black. The circles indicate female subjects and the squares male subjects.

In 8 trios we found higher-than-normal range serum-induced C5b-9 formation, both in the probands (remission, n=8) and in their fathers (proband: 194 ± 26%; father: 197 ± 60%, **Figure 5A**). In contrast, the serum samples from the mothers caused normal C5b-9 formation on endothelial cells (117 ± 19%, **Figure 5A**). These results suggest that the probands inherited a genetic defect from the father. A representative trio is shown in **Figure 5D** (see clinical history in **Supplementary Material**).

In 4 trios the sera from both the probands (remission, n=3; acute, n=1) and their mothers induced excessive C5b-9 formation on HMEC-1 (patient: 229 ± 51%; mother: 181 ± 22%; **Figure 5B**) indicating a genetic abnormality inherited from the mother. Consistent with this, in these trios the serum from the fathers induced normal C5b-9 formation on endothelial cells (94 ± 19%; **Figure 5B**). A representative trio is shown in **Figure 5E** (see clinical history in **Supplementary Material**).

Finally, in 5 trios both parents exhibited normal C5b-9 formation on ADP-activated HMEC-1, whereas the test was positive in all probands (remission, n=5) (patient: 237 ± 110%; father: 116 ± 15%; mother: 134 ± 12%, **Figure 5C**). For these trios, the results of the test did not allow us to determine whether the disease was associated with a *de novo* genetic abnormality, a recessive inheritance, or whether it was not genetically determined.

## Discussion

By studying 223 subjects who belong to 60 aHUS pedigrees, here we show that the ex vivo assay based on serum-induced C5b-9 formation on activated HMEC-1 may represent a potential tool for a rapid evaluation of the functional impact of rare complement gene variants. In addition, we provide evidence that this assay could also be useful for uncovering inherited complement dysregulation in aHUS patients who do not carry variants in known disease-associated genes.

Genetic analysis using next-generation sequencing makes it possible to search for nucleotide variants in the whole complement gene set quickly and inexpensively. Nevertheless, some particularities of complement genes should be considered, including the high sequence homology between *CFH* and *CFHRs* genes (9), and additional techniques, such as MLPA, are often required to correctly identify the generation of structural variations in the Regulators of Complement Activation (RCA) gene cluster (27). Among the variants identified through genetic analyses in aHUS patients, only a subgroup can be defined as pathogenic with certainty, based on either published functional studies or a clear-cut effect on transcription/translation (9, 28). But we do not know the functional significance of a large majority of variants. This is an important issue, because knowing the real functional impact of gene variants is crucial to better understanding the pathogenesis of aHUS in each patient and can help confirm a diagnosis, guide patient management, inform prognosis, and reduce the need for invasive procedures (8, 9, 28, 29).

It is not easy to determine the functional meaning of a gene variant. In line with published guidelines (13) (https://www.acgs.uk.com/media/11631/uk-practice-guidelines-for-variant-classification-v4-01-2020.pdf), rare gene variants can be categorised – as pathogenic, likely pathogenic, variants of uncertain significance, likely benign and benign – based on criteria that include prevalence in healthy individuals (MAF), localisation within the gene and segregation with the disease, as well as in silico estimation of the influence of the variant on protein structure or function.

Recently, the capacity of in silico predictions to really establish whether or not a variant may be pathogenetic has been doubted, and this limitation applies particularly to gain-of-function variants in *C3* and *CFB* genes (30, 31). Even the CADD tool, which scores the predicted deleteriousness of variants by integrating multiple annotations into one metric, often fails. In this regard, a study by Merinero et al. (32) described the functional characterisation of 28 new *CFH* gene variants. All 17 variants that were classified as pathogenic in silico were confirmed to be pathogenic in functional studies. However, among the 11 variants categorised as benign or VUS, 7 were found to be pathogenetic by functional studies, leading the authors to conclude that with the current algorithms’ prediction is unreliable, mainly in the case of variants categorised as benign or VUS. In a subsequent paper the same group further demonstrated the limitations of algorithms in predicting the pathogenicity of 105 *CFH* variants, concluding that functional studies are necessary (29).

Functional studies are complicated and time-consuming. In addition, each complement factor and regulator carriers out multiple and complex activities and several functional tests should be performed to obtain a clear picture of the impact of a given variant. In this regard, only 29 of the 79 VUS studied by Merinero et al. (29) turned out to alter FH expression or function as observed in 4 different functional *in vitro* tests. However, one cannot rule out the possibility that for any of the remaining variants, additional functional tests would have revealed a pathogenic impact on FH function.

With the goal of overcoming the above obstacles, here we decided to use the ex vivo serum-induced C5b-9 formation test on activated endothelium (10). Consistent with our published results (6, 10) the assay was always positive with serum from aHUS patients who were in remission, and it could not discriminate in patients between carriers and non-carriers of complement gene variants.

This finding may be related both to the presence of additional predisposing factors (such as the CFH H3 haplotypes) and to subclinical chronic disease activity associated with the presence in aHUS serum of heme, extracellular vesicles, ROS, or inflammatory cytokines that may favour the formation of C5b-9 on an activated endothelium (33–35).

We therefore changed our approach, based on two considerations. First, penetrance of aHUS is incomplete, as documented by the finding in aHUS families of numerous asymptomatic relatives who carry the same gene abnormality as the proband (24). In addition, the large majority of aHUS disease-associated variants are inherited from an unaffected parent (2, 36). Second, by studying a small number of relatives of aHUS patients, we previously found that sera from unaffected relatives carrying the same pathogenic variant as the proband caused higher-than-normal C5b-9 formation on endothelium (10), whereas sera from non-carrier relatives did not.

With these concepts in mind, and to overcome the confounding effect of aHUS status, here we performed the C5b-9 formation test in several aHUS pedigrees with at least one unaffected relative who shared with the proband a published functionally characterised variant. We found that almost 93% of unaffected relatives with pathogenic variants in circulating complement factors exhibited a positive serum-induced C5b-9 formation test, evidence of the test’s high sensitivity in identifying functional variants. The test in relatives showed that all non-carriers exhibited a negative C5b-9 formation test. In addition, all the unaffected relatives with rare complement gene variants that did not segregate with aHUS exhibited a negative serum-induced C5b-9 formation, indicating that the test was specific for identifying disease-relevant gene abnormalities. Based on the above results we then investigated whether the ex vivo C5b-9 formation test could be useful for better and more quickly characterising LPV, VUS and likely benign/benign variants.

Sera from all relatives but one who carry LPV in known disease-associated genes induced higher-than-normal C5b-9 formation. The *CFH* p.1182delins LPV was described previously in a family (37). Although no functional studies were performed, the variant may have profound effects on FH activity, since it causes the insertion of 12 extra amino acids in the SCR20, a domain that encompasses binding sites for C3b and polyanionic carbohydrates and is important for complement regulation on host surfaces (38–40). The *CFH* p.896_900 del and the p.W978R variants are located in SCR15 and SCR16, respectively, which belong to the central segment of the protein whose function is still poorly characterised (41, 42). As for the LPVs in C3 gene, the functional C5b-9 test was positive in carriers of the p.D61N. This is in line with its location in the MG1 domain, which participates in the formation of a key ring and may be crucial for the C3 structure–function relationships (43). The finding that serum from the unaffected carrier of the *C3* p.G321V did not induce excessive C5b-9 formation on HMEC-1 suggested that this LPV does not have a functional effect.

The results from serum of the carrier of the LPV in the putative candidate *CFHR5* gene may help to clarify the involvement of FHR5 in aHUS. Several authors have described potentially pathogenic *CFHR5* variants in aHUS patients (44–48). On the other hand, Osborne et al. (30) failed to find a significant association between rare variants in *CFHR5* and aHUS. Among the FHR proteins, only FHR5, like FH, has FI-dependent cofactor activity, leading to C3b inactivation, and C3 convertase decay accelerating activity (48), but these activities have been observed in the fluid phase only at higher than physiological concentrations (49, 50). On the other hand, FHR5 has been shown to specifically interact with C3b, heparin and other cellular and extracellular ligands through its central SCR3-7, suggesting it may carry out local cell and tissue activity (48). The present data showing that serum from the subject carrying the *CFHR5* p.E163Kfs10x variant – which causes protein truncation in SCR3 – induced excessive C5b-9 formation on the endothelial cell surface, supports the hypothesis that FHR5 might regulate complement at the endothelial cell surface.

Notably, all relatives with VUS in aHUS-associated genes had a positive C5b-9 formation test, whereas the assay was negative for carriers of VUS only in putative candidate genes (*CFHR4*, and *C5)*. The latter results do not support the hypothesis that VUS we found in candidate genes play a role in inducing complement dysregulation on endothelium. However, other functional studies should be performed before completely ruling out the possibility that these VUS have a role in aHUS pathogenesis.

Consistent with the previous report by Merinero et al. (32) that documented the pathogenicity of 4 *CFH* variants categorised as benign, the unaffected relative from our cohort who carries a likely benign *CFH* variant was positive in the C5b-9 formation test. Altogether, the present results and other published data (29, 32) confirm that in silico predictions might be unreliable in the case of benign or likely benign variants.

The C5b-9 assay in parents was particularly helpful in defining the relative functional effect of rare variants in pedigrees in which the proband carried more than one genetic abnormality. Thus, in two pedigrees both variants that the proband inherited from either parent were associated with ex vivo complement activation on the endothelium, whereas in two other pedigrees only one variant appeared to be functional. Notably, in one of the latter pedigrees the proband inherited a *C3* LPV from the father and a likely benign variant in *CFH* from the mother. The test reversed the in silico prediction; indeed, serum from the mother was positive in the C5b-9 test, while a negative result was found with serum from the father with the LPV.

Finally, the C5b-9 test allowed us to unmask in a few patients without rare variants – either in disease-associated or in candidate genes – a genetic liability inherited from unaffected parents. This applied to pedigrees in which either the father or the mother had a positive test, revealing the presence of a genetic abnormality predisposing to complement dysregulation. In one pedigree, the maternal inheritance of the unknown genetic factor was confirmed by the positive test in the maternal aunt.

Exome sequencing has proven to be a rapid and cost-effective way of discovering causative genes and has been useful in identifying mutations involved in rare Mendelian diseases (51). However, pitfalls and hurdles remain, including the difficulty of selecting from among a huge number of rare, potentially causative variants in several genes. This issue is further amplified by the oligogenic nature and incomplete penetrance of aHUS, particularly in non-familial cases. Combining exome sequencing with results from C5b-9 tests in parents as well as in informative, unaffected relatives could help researchers select variants and possibly identify new genetic factors that predispose to aHUS.

The C5b-9 formation test in unaffected relatives described here has some limitations that should be considered. First, while the assay catches abnormalities in soluble complement proteins that are present in serum, it is not informative regarding the functional effects of rare variants in complement regulatory proteins expressed on the cell membrane, such MCP or THBD, since HMEC-1 cells express the wild-type proteins. To overcome this limitation, we are working to obtain endothelial cells from iPSC derived from PBMC from patients and unaffected relatives with *MCP* or *THBD* variants that will be used as target cells in the C5b-9 formation test. In addition, the test was not conclusive in pedigrees without identified variants with negative C5b-9 results in both parents. In these cases, the question of whether the disease in the proband was associated with a *de novo* genetic abnormality or a recessive inheritance or was not genetically determined remained open.

In conclusion, the results of this study provide evidence that the serum-induced C5b-9 formation test performed in unaffected relatives of aHUS patients may be a tool for rapidly obtaining important information about the functional consequences of rare complement gene variants. When combined with exome sequencing the assay might be of help in guiding variant selection, to identify new aHUS-associated genetic factors. Genetic studies in rare diseases are often complicated by incomplete penetrance. In addition, the progressive decrease of average size of families, at least in Europe and Western countries, lowers the probability to find familial cases. The approach of using disease-relevant assays in relatives to extend the number of informative subjects, could be applied to other rare diseases to help variant selection and characterization, while limiting the hurdle of recruiting large cohorts of patients.

## Data availability statement

The original contributions presented in the study are included in the article/**Supplementary Materials**. Further inquiries can be directed to the corresponding author.

## Ethics statement

The studies involving human participants were reviewed and approved by Ethical Committee of the Azienda Sanitaria Locale Bergamo, Italy. Written informed consent to participate in this study was provided by the participants’ legal guardian/next of kin.

## Author contributions

SG, MG, MB, SA, and MN designed the research, interpreted the data, and wrote the paper; SG, RP, MA, LL, CM, MB, and DS performed the research and analysed the data; EB provided detailed clinical information on patients and wrote family histories; MN, SA, AB, and GR critically revised the manuscript. All authors contributed to the article and approved the submitted version.
