# Supplementary material for: An ex vivo test to investigate genetic factors conferring susceptibility to atypical haemolytic uremic syndrome
Source: Front Immunol. 2023 Feb 9;14:1112257. doi: 10.3389/fimmu.2023.1112257 (PMC9949374; doi:10.3389/fimmu.2023.1112257)
Supplement: Supplementary file 1 [file DataSheet_1.docx]

**An ex vivo test to investigate genetic factors conferring susceptibility to atypical haemolytic uremic syndrome**

Sara Gastoldi^1^, Sistiana Aiello^1^, Miriam Galbusera^1^, Matteo Breno^1^, Marta Alberti^1^, Elena Bresin^1^, Caterina Mele^1^, Rossella Piras^1^, Lucia Liguori^1^, Donata Santarsiero^1^, Ariela Benigni^1^,

Giuseppe Remuzzi^1^ and Marina Noris^1^.

**Corresponding author**:

Marina Noris, PhD

Istituto di Ricerche Farmacologiche Mario Negri IRCCS

Via Camozzi 3, 24020 Ranica Bergamo, Italy.

Phone:+39 035 4535362

Fax: +39 035 4535371

email: marina.noris@marionegri.it

**SUPPLEMENTARY MATERIAL:**

- Family history of patients described in the figures
- Supplementary tables
- Supplementary figures

***Family history of patients described in the figures***

**Figure 2**

**Family #267 (patients #871 and #875**):

This is a family from Argentina that was referred to our International Registry of Recurrent and Familial HUS/TTP in 2005 because of familial history of aHUS.

The proband (patient #875) is a male who developed aHUS at 19 years of age, after upper respiratory tract infection and vomiting, with severe renal failure (s-creatinine 6 mg/dl) reaching end stage renal disease (ESRD) soon after disease onset, despite treatment with plasma exchange (PEX) and infusions. C3 and C4 levels were in normal the range and ADAMTS13 activity was slightly reduced (47%). At last follow-up the patient was on regular dialysis treatment.

The brother (patient #871) had his first acute episode of aHUS at 23 years of age, with haemolytic anemia, thrombocytopenia and acute kidney injury (AKI). After treatment with plasmapheresis, fresh frozen plasma and steroids, he partially recovered but three months later he had a disease relapse. He received the same treatment obtaining hematological remission but renal function did not improve. C3 and C4 levels were in the normal range. ADAMTS13 activity was normal (77%). At the last follow-up he was in hematological remission but with chronic kidney disease.

The maternal family history includes a male cousin (patient #872) who experienced aHUS onset at 10 years of age and a disease relapse at 14 years reaching ESRD as final outcome.

These patients did not receive eculizumab treatment since they manifested aHUS and disease relapses years before eculizumab approval.

The genetic screening of the patients revealed the presence in heterozygosis of the known pathogenic variant *p.S1191L* in *CFH* gene, already described in literature and found also in Family #265. This variant segregates in the family with the disease, with incomplete penetrance, as shown in Figure 2B.

**Family #1245 (patient #2037).**

Patient #2037 (already described by 'Galbusera M. et al. *AJKD* 2019') manifested aHUS at the age of 48 years with thrombocytopenia, haemolytic anaemia and AKI. The patient received several sessions of PEX but did not achieve remission and remained dialysis-dependent. One month after disease onset the patient started eculizumab. Hematological parameters normalized and C5b-9 deposits on ADP-activated and unstimulated HMEC-1 fell to the lower limit of normal range, but renal function did not recover. After 10 months of standard maintenance of eculizumab, treatment was progressively spaced to every 3-, 4- and 5-weeks. Until 4-week dose spacing, clinical parameters remained stable and serum-induced C5b-9 deposits were in the lower normal range. At 5-week interval between eculizumab doses, the patient experienced a drop in haptoglobin levels to 9 mg/dl and LDH increased to 513 IU/L, which was associated with increase of ex-vivo C5b-9 endothelial deposits largely over upper limit of normal range. Return to eculizumab every 4-weeks resulted in normalization of haematological parameters and of complement deposits. S-creatinine progressively decreased although patient still needs dialysis every 5 days.

Family history was negative for aHUS. At genetic screening the patient was found heterozygous for a known pathogenic abnormality, the reverse hybrid *CFHR1/CFH* gene. In the family there are three unaffected carriers, consistent with an incomplete penetrance (Figure 2C).

**Figure 4**

**Family #052 (patient #347)**

Patient #347 is an Italian male who was hospitalized at the age of 6 months because of irritability, anorexia, fever and anuria. At admission, laboratory exams showed anaemia (Hb 8.3 g/dl), thrombocytopenia (platelets 58,000/μl), acute renal failure (s-creatinine 1.6 mg/dl), high levels of LDH (4150 IU/L), undetectable haptoglobin, presence of schistocytes in the peripheral blood smear, low levels of C3 (44 mg/dl) and normal ADAMTS13 activity (99%), consistent with a diagnosis of aHUS. The child underwent hemodialysis and received five transfusions of packed red blood cells. Due to appearance of oedema in the lower limbs and increase in blood pressure (PA 165/98 mmHg), the child was treated with antihypertensive therapy. The biochemical assays also showed hypoprotidemia and proteinuria, thus Solumedrol bolus therapy was started for three days, followed by oral steroid with slow reduction of the oedema. The patient was discharged after 40 days of hospitalization with persistence of nephrotic syndrome and hypertension.

After one month the child manifested a relapse of aHUS with worsening of renal function (s-creatinine 2.6 mg/dl, oliguria with proteinuria and haematuria), and anemia. For the persistence of oliguria, therapy with dopamine and furosemide was started intravenously, obtaining a transient increase in diuresis. Subsequently, plasma infusion, transfusion of concentrated red cells and a session of hemodialysis were performed. After this session, hypertensive crisis appeared, with left heart failure and acute pulmonary oedema (probably secondary to renal stimulation due to rapid volume subtraction during haemodialysis), and tracheal intubation was performed for three days. Meanwhile, he continued with blood transfusions. After one month, clinical conditions improved and the child was discharged under antihypertensive therapy, however with persistence of renal insufficiency (s-creatinine 1.8 mg/dl), oliguria and proteinuria.

Another aHUS relapse was documented after one year, with worsening of renal function, severe anaemia and high levels of LDH (1400 IU/L), and the child was again treated with plasma infusion, transfusion of concentrated red cells and hemodialysis obtaining again hematological remission, but renal function was irreparably compromised. At last follow-up at the age of 9 years, the patient was close to ESRD (s-creatinine 5 mg/dl).

The patient did not receive eculizumab treatment since he manifested aHUS and disease relapses years before eculizumab approval.

Family history was negative for aHUS. Genetic screening revealed a heterozygosity for a known pathogenic variant *p.V1197A* in *CFH* gene, inherited from the father and also a heterozygous rare *LPV* in *CFH* gene, *p.W978R*, inherited from the mother.

**Family #390 (patient #1052)**

Patient #1052 developed aHUS at 23 years of age, one week after appearance of fever, vomiting, oliguria and severe arterial hypertension. At hospital admission the exams showed acute severe renal insufficiency (s-creatinine 6 mg/dl), anaemia (Hb 7.3 g/dl), thrombocytopenia (plt 73,000/µl), increased LDH (3257 IU/L), presence of schistocytes. ADAMTS13 activity was normal (100%). Diagnosis of aHUS was hypothesized, the patient started immediately daily hemodialysis and multi-drug antihypertensive treatment, and received plasma exchange with normalization of platelet count. She underwent kidney biopsy which showed thrombotic microangiopathy, glomerular sclerosis, chronic inflammatory granulomatous infiltration in the interstitium. The patient continued plasma-exchange and was treated also with Vincristine due to persistence of anaemia and haemolysis, but could stop dialysis treatment due to improvement of renal function (s-creatinine 2.8 mg/dl). The patient was discharged after one month, in good haematological conditions (normalization of platelet count, rise in haemoglobin levels), with normal ADAMTS13 activity (55%), but with chronic kidney disease.

However some months later the patient manifested a disease relapse with haemolysis, severe renal insufficiency needing again daily hemodialysis, severe arterial hypertension not responsive to multidrug antihypertensive therapy. She underwent bilateral nephrectomy and also splenectomy, but due to severe complications of a pneumonia with sepsis the patient died.

The patient did not receive eculizumab treatment since she manifested aHUS and disease relapse years before the eculizumab approval.

Family history was negative for aHUS. Genetic screening revealed a heterozygous for a known pathogenic variant *c.3493+1G>A* in *CFH* gene, inherited from the mother and also found heterozygous for a rare *VUS* (variant of unknown significance) in *CFH* gene, *p.N516K*, inherited from the father.

**Family #1485 (patient #2314)**

Patient #2314 is an Italian child who at 6 years of age presented with anuria for over 3 weeks and the need of dialysis for at least one month. At admission, laboratory exams showed acute renal failure (s-creatinine 2.47 mg/dl), thrombocytopenia (platelets 86,000/μl), high levels of LDH (4856 IU/L), undetectable haptoglobin, hypocomplementemia C3 (50 mg/dl). He was treated with antibiotic therapy, steroids, daily plasma infusions, red blood cell transfusions, antihypertensive therapy, and dialysis, with slow resumption of diuresis but with persistence of nephrotic proteinuria (P/Cr 10 mg/mg) and haematuria. During dialysis the child had resumption of platelet consumption and haemolysis, associated with arterial hypertension. Thus, two weeks after HUS onset, treatment with 300 mg eculizumab (every week for four weeks) was started, and Metilprednisolone bolus therapy was also performed for three days followed by oral steroid.

After 40 days of hospitalization, the patient was discharged under eculizumab treatment (300 mg every 2 weeks) with haematological remission but with persistence of nephrotic syndrome, renal insufficiency and hypocomplementemia C3. One year later the child was still under eculizumab therapy without flare-up of microangiopathy but with chronic proteinuric nephropathy.

At the genetic screening the patient was found heterozygous for a rare *VUS* in *C3* gene, *c.74+1delG*, inherited from the father, and also heterozygous for a rare *VUS* in *CFB* gene, *p.N340Y*, inherited from the mother.

**Family #265 (patients #870 and #868)**

Patient #870 (already described by Saland JM et al. *cjasn* 2009) is a Caucasian male who presented at the age of 9 months with renal insufficiency (s-creatinine 2.7 mg/dl), haemolytic anaemia and thrombocytopenia without diarrhea, sepsis, or signs of infection. At that time, the maternal family history included two female second cousins who developed end stage renal disease (ESRD) secondary to thrombotic microangiopathy (TMA). Subsequent to the child's presentation, a third female maternal second cousin also developed aHUS that precipitated to ESRD. Of these relatives, one (patient #868) failed isolated kidney transplantation due to aHUS recurrence, one was dialysis-dependent, and the other one deceased due to complications of ESRD.

The proband underwent renal biopsy, which showed TMA with acute tubular necrosis, and moderate chronic interstitial nephritis with focal fibrosis. Several weeks of plasmapheresis resulted in resolution of haemolysis and improvement in s-creatinine to 0.6 mg/dl.

Subsequently, the child suffered from several recurrences of aHUS, most of them preceded by viral infections or catheter-related bacterial infections, and each treated with plasma exchanges. However, despite a prophylactic regimen of twice weekly plasma exchange was started at the age of 3 years, renal function declined significantly with inexorable progression to ESRD. ADAMTS13 activity was in the normal range (89%).

At the age of 4 years, the child received a combined split liver-kidney transplant (LKT) with pre-operative plasma exchange and enoxaparin anticoagulation. Both grafts had excellent immediate function. No plasma infusions or plasma exchanges were given post-operatively.

Two years post-transplant, grafts function was maintained and child’s health was generally good. At the last follow-up, at the age of 9 years the patient was still in good clinical conditions.

The patient did not receive eculizumab treatment since he manifested aHUS and disease relapses, and also underwent LKT, before eculizumab approval.

At the genetic screening the patient was found heterozygous for a known pathogenic variant in *CFH* gene, *p.S1191L*, inherited from the mother and present also in the maternal affected relative #868, but subsequently he was also found heterozygous for a rare *VUS* in *CFI* gene, *p.E554V*, inherited from the mother but absent in the maternal affected relative #868.

**Family #2880 (patient #4050)**

Patient #4050 is an Italian female who developed aHUS at 12 years of age, with severe acute renal failure, oligo-anuria and arterial hypertension, thrombocytopenia and moderate anaemia, preceded by STEC negative diarrhea, vomiting and fever. At admission, laboratory exams showed s-creatinine 9 mg/dl, platelets 58,000/μl, high levels of LDH, undetectable haptoglobin, reduced levels of C3 (65 mg/dl) with normal C4; ADAMTS13 activity was normal. She was treated with antibiotic and antihypertensive therapy, one transfusion of red blood cells, haemodialysis for 8 days, obtaining normalization of haematological parameters and progressive improvement of renal function. One month after the HUS episode the patient reached complete remission, with only persistence of reduced C3 levels (54 mg/dl).

Family history was negative for aHUS. At the genetic screening the patient was found heterozygous for a *LPV* in *C3* gene, *c.962G>T (p.G321V)*, inherited from the father #4051, and also heterozygous for a *LBV* in *CFH* gene, *c.2957-7A>G*, inherited from the mother #4052.

**Family #412 (patient #1070)**

Patient #1070 is a caucasian female who developed aHUS at 1 year of age, with acute renal failure and anuria for 20 days, anaemia and thrombocytopenia, preceded by STEC negative diarrhea and vomiting. At admission, laboratory exams showed s-creatinine 3.97 mg/dl, platelets 58,000/μl, LDH 3192 IU/L, presence of schistocytes in the peripheral blood smear, reduced levels of C3 (68 mg/dl). She manifested also severe arterial hypertension and neurological signs (convulsions). She was treated with antibiotic therapy, antihypertensive therapy, red blood cell transfusions, plasma exchange, and dialysis for 34 days, with normalization of haematological parameters but persistence of hypertension and renal impairment. ADAMTS13 activity was normal (73%).

The patient did not receive eculizumab treatment since she manifested aHUS before the eculizumab approval.

Family history was negative for aHUS. At the genetic screening the patient was found heterozygous for a *LPV* in CFHR5 gene, *c.485_486dupAA (p.E163Kfs10x)*, inherited from the mother #2583, and heterozygous for a benign variant in *CFHR2* gene, *c.325A>G (p.T109A)*, inherited from the father #2654.

**Figure 5**

**Family #1124 (patient #1897)**

The proband (patient #1897) is a male who developed aHUS at 1 year of age, with AKI and anuria, anemia and thrombocytopenia, preceded by STEC negative diarrhea. The child was treated at onset with continuous venovenous haemofiltration (CVVH) and eculizumab with progressive improvement of clinical conditions and complete normalization of renal function. After 3 weeks he stopped haemodialysis treatment and after 2 months also eculizumab therapy was discontinued.

C5b-9 deposits on ADP-activated HMEC-1 were firstly evaluated one month after eculizumab discontinuation with normal finding (89%), but the subsequent tests carried out after two years showed persistently elevated C5b-9 deposits (range: 155-334%). C5b-9 deposits on ADP-activated HMEC-1 were found higher than normal also in the father, suggesting complement-associated disease pathogenesis. At last follow-up at 7 years of age, the patient was in complete remission without aHUS relapse.

Family history was negative for aHUS. Search for complement gene variants, CFH/CFHR rearrangements and anti-CFH autoantibodies was negative in this patient, but elevated C5b-9 deposits on unstimulated and ADP-activated HMEC-1 in the patient in acute phase pre-eculizumab, and increased C5b-9 deposits in remission on ADP-activated HMEC-1 in the patient and also in the father, indicated complement-dependent disease pathogenesis. (Figure 5D).

**Family #1998 (patient #3023)**

The proband (patient #3023) is a female who developed aHUS at 25 years of age, one month after appearance of periorbital and lower limbs oedema with anasarca, headache. At hospital admission the exams showed severe renal insufficiency (s-creatinine 11 mg/dl) with proteinuria (1.8 g/24h) and severe arterial hypertension, anaemia (Hb 7.9 g/dl), thrombocytopenia (plt 92,000/µl), increased LDH (724 IU/L), undetectable haptoglobin, reduced levels of C3 (72 mg/dl), normal ADAMTS13 activity (65%). The diagnosis of aHUS was done, the patient started immediately haemodialysis and received two packed red cells transfusions and antihypertensive therapy. She was also treated with eculizumab but without recovery of renal function, thus after 5 infusions the treatment was stopped.

The patient was discharged in ESRD with signs of chronicity (small sclerotic kidneys) needing chronic haemodialysis but with normal values of arterial pressure under antihypertensive therapy.

After one year the patient underwent successful donor-related kidney transplant (from the mother), with eculizumab prophylaxis, and at the last follow-up, 18 months later, the patient was still in good condition with normal renal function (s-creatinine 0.9) without rejection or aHUS relapse and under maintenance treatment with eculizumab every 21 days.

Family history was negative for aHUS. Search for complement gene variants, CFH/CFHR rearrangements and anti-CFH autoantibodies was negative in this patient, but elevated C5b-9 deposits on unstimulated and ADP-activated HMEC-1 in the patient in acute phase pre-eculizumab, and also in the mother and in the maternal aunt, on ADP-activated HMEC-1, indicated complement-dependent disease pathogenesis. (Figure 5E).

**Supplementary table 1. Characteristics of aHUS Patients.**

| ***Fam code*** | ***DNA code*** | ***Proband/***  ***affected*** | ***sex*** | ***spor/rec*** | ***Family history*** | ***Age of onset*** | ***Phase*** | ***serum C3 levels*** | ***plasma sC5b9 levels*** | ***C5b9 deposition (%)*** | ***Variants*** | ***CFH H3 haplotype*** | ***MCP haplotype*** | ***CFHR1*Bhaplotype*** |
| --- | --- | --- | --- | --- | --- | --- | --- | --- | --- | --- | --- | --- | --- | --- |
| #20 | 214 | proband | F | spor | no | 31 | acute | 122 | 375 | 309% | PV-CFH + VUS CFHR4 | 0 | 1 | 1 |
|  |  |  |  |  |  |  | rem | 105 | 233 | 383% |  |  |  |  |
| #101 | 499 | proband | F | rec | no | 21 | acute | 55 | 381 | 306% | PV-CFH + LB CFHR4 | 0 | 1 | 1 |
|  |  |  |  |  |  |  | rem | 51 | 725 | 195% |  |  |  |  |
|  |  |  |  |  |  |  | 2° rem |  |  | 193% |  |  |  |  |
| #267 | 875 | proband | M | spor | yes | 19 | rem | 115 | 447 | 504% | PV-CFH + LB C5 | 1 | 0 | 1 |
|  | 871 | affected | M | rec | yes | 23 | rem | 109 | 656 | 468% | PV-CFH + LB C5 | 1 | 0 | 1 |
| #271 | 882 | proband | M | spor | no | 1 | rem | 136 | 1100 | 209% | no | 0 | 1 | - |
| #380 | 1005 | proband | M | spor | no | 2 | rem | 67 | 128 | 213% | PV-Hybrid + VUS C3 | 0 | 2 | - |
| #551 | 1213 | proband | M | rec | yes | 48 | rem | 111 | 236 | 605% | PV-Hybrid | 1 | 2 | - |
|  | 1695 | affected | F | spor | yes | 20 | acute | 79 | 329 | 303% | PV-Hybrid | 0 | 1 | - |
| #1065 | 1838 | proband | M | spor | no | 2 | acute | 75 | 497 | 295% | PV-MCP | 0 | 2 | - |
| #1245 | 2037 | proband | M | spor | no | 48 | acute | 81 |  | 235% | PV-Hybrid | 0 | 1 | - |
| #2169 | 3244 | proband | F | spor | no | 32 | acute | 67 |  | 222% | LPV C3 | 2 | 1 | 2 |
|  |  |  |  |  |  |  | rem | 48 |  | 154% |  |  |  |  |
| #2813 | 3955 | proband | M | spor | no | 6 | rem | 79 |  | 123% | VUS CFH | 0 | 0 | 0 |
| **#2816** | **3961** | **proband** | **F** | **rec** | **yes** | **4** | **rem** | **121** |  | **232%** | **PV-MCP** | **1** | **1** | **1** |
|  | **3960** | **affected** | **M** | **spor** | **yes** | **4** | **rem** | **97** |  | **210%** | **PV-MCP** | **1** | **1** | **1** |
| #2880 | 4050 | proband | F | spor | no | 12 | acute | 65 |  | 207% | LB CFH + LPV C3 | 0 | 0 | 2 |
|  |  |  |  |  |  |  | rem | 54 |  | 181% |  |  |  |  |
|  |  |  |  |  |  |  | 2° rem |  |  | 202% |  |  |  |  |
| #1494 | 2318 | proband | F | spor | no | 1 | rem | 94 |  | 245% | VUS CFHR4 | 0 | 0 | - |
|  |  |  |  |  |  |  | 2° rem |  |  | 190% |  |  |  |  |
| #1538 | 2335 | proband | F | spor | no | 1 | rem | 82 |  | 185% | no | 0 | 0 | - |
| #1544 | 2337 | proband | F | spor | no | 2 | rem | 102 |  | 200% | no | 1 | 1 | - |
|  |  |  |  |  |  |  | 2° rem |  |  | 246% |  |  |  |  |
| #1650 | 2505 | proband | F | spor | no | 3 | rem | 93 |  | 181% | no | 0 | 2 | - |
| #1785 | 2751 | proband | F | spor | no | 28 | rem | 62 |  | 223% | no | 1 | 1 | - |
| #1998 | 3023 | proband | F | spor | no | 25 | acute | 72 |  | 208% | no | 1 | 1 | - |
| #2071 | 3116 | proband | F | rec | no | 18 | rem | 73 |  | 218% | VUS C5 | 0 | 1 | 1 |
|  |  |  |  |  |  |  | 2° rem |  |  | 171% |  |  |  |  |
| #84 | 444 | proband | F | spor | no | 0.8 | rem | 74 |  | 165% | no | 0 | 1 | 0 |
| #418 | 1074 | proband | F | rec | no | 2.5 | rem | 106 |  | 232% | no | 1 | 1 | 2 |
| #1124 | 1897 | proband | M | spor | no | 1 | rem | 110 | 310 | 186% | no | 1 | 0 | - |
| #1279 | 2074 | proband | M | spor | yes | 0.6 | rem | 88 |  | 388% | VUS CFHR4 | 1 | 1 | 0 |
| #2815 | 3958 | proband | F | spor | no | 2 | rem | 82 |  | 165% | no | 0 | 0 | 1 |
|  |  |  |  |  |  |  | 2° rem |  |  | 274% |  |  |  |  |
| #45 | 370 | proband | M | spor | yes | 0.8 | acute | 151 |  | 246% | PV-CFH | 1 | 2 | 1 |
| #120 | 543 | proband | F | rec | no | - | rem | 75 |  | 196% | PV-CFH | 2 | 1 | - |
| #155 | 592 | proband | F | rec | no | 0.8 | rem |  |  | 203% | VUS CFH | 1 | 1 | - |
| #176 | 617 | proband | F | rec | yes | 20 | rem | 82 |  | 192% | PV-Hybrid | 0 | 2 | - |
| #194 | 652 | proband | F | rec | yes | 1 | rem | 127 | 342 | 200% | LPV CFH | 1 | 1 | - |
| #251 | 849 | proband | M | spor | no | 1 | acute | 151 |  | 191% | PV-CFH | 1 | 1 | - |
| #265 | 870 | proband | M | rec | yes | 0.9 | rem | 110 |  | 168% | PV-CFH + VUS CFI | 0 | 0 | - |
|  | 868 | affected | F | spor | yes | - | rem | 123 |  | 233% | PV-CFH | 0 | 1 | - |
| #390 | 1052 | proband | F | spor | no | 23 | rem | 124 |  | 211% | PV-CFH + VUS CFH | 2 | 1 | 2 |
| #603 | 1305 | proband | M | rec | no | 0.8 | ecu |  |  | 123% | PV-CFH | 1 | 1 | 1 |
| #856 | 1591 | proband | M | rec | no | 35 | acute | 79 | 421 | 474% | PV-CFH + VUS CFB | 1 | 2 | 2 |
| #181 | 628 | proband | M | rec | no | 0.6 | acute | 58 |  | 183% | LPV CFH | - | 2 | - |
| #52 | 347 | proband | M | rec | no | 0.6 | acute | 55 |  | 221% | PV-CFH + LPV CFH | 0 | 0 | - |
| #210 | 682 | proband | M | rec | yes | 1 | rem | 72 |  | 285% | PV-CFH | 0 | 1 | - |
| #563 | 1238 | proband | F | spor | no | 18 | rem | 39 | 1436 | 236% | PV-C3 + VUS CFHR4 | 2 | 2 | - |
| **#458** | **1114** | **proband** | **M** | **rec** | **no** | **13** | **rem** | **118** |  | **176%** | **PV-MCP** | **0** | **2** | **0** |
| #158 | 591 | proband | M | rec | yes | 8 | ecu | 33 |  | 84% | PV-C3 | 1 | 1 | - |
|  | 2501 | affected | M | spor | yes | 49 | ecu | 38 |  | 96% | PV-C3 | 0 | 2 | - |
| #283 | 899 | proband | M | spor | no | 27 | rem | 78 |  | 223% | no | 0 | - | - |
| #316 | 931 | proband | F | rec | yes | 0.6 | acute | 98 |  | 292% | PV-CFHR1 | 2 | 0 | - |
| #412 | 1070 | proband | F | spor | no | 1 | rem | 133 |  | 183% | LPV CFHR5 + B CFHR2 | 0 | 1 | 2 |
| **#646** | **1342** | **proband** | **M** | **rec** | **no** | **32** | **rem** | **114** | **251** | **464%** | **PV-MCP + LB CFI** | **1** | **2** | **1** |
| #1237 | 2034 | proband | M | spor | no | 1.7 | rem |  | 245 | 303% | no | 1 | 0 | - |
| #1485 | 2314 | proband | M | spor | no | 6 | ecu | 61 |  | 94% | VUS C3 + VUS CFB | 0 | 1 | - |
| #473 | 1136 | proband | F | spor | no | 26 | rem | 80 |  | 160% | no | 2 | - | - |
| #623 | 1331 | proband | F | spor | no | 5 | rem | 71 |  | 154% | B C5 | 0 | 1 | - |
| #1509 | 2315 | proband | F | spor | no | 41 | rem | 95 |  | 429% | no | - | - | - |
| **#782** | **1503** | **proband** | **F** | **spor** | **yes** | **35** | **rem** | **96** |  | **173%** | **PV-MCP** | **1** | **1** | - |
| #1077 | 1827 | proband | M | spor | no | 8 | acute | 123 | 392 | 342% | VUS C3 | 0 | 0 | 2 |
| #334 | 951 | proband | M | spor | no | 1 | rem | 82 |  | 186% | no | 2 | 1 | - |
| #308 | 926 | proband | M | spor | no | 1 | rem | 88 |  | 219% | no | 1 | - | - |
| #81 | 453 | proband | F | spor | yes | 1 | rem | 97 |  | 234% | PV-CFH | - | 2 | - |
| #1683 | 2577 | proband | M | spor | no | 1 | rem | 113 |  | 204% | VUS C5 | 1 | 2 | - |
| #4 | 170 | proband | F | rec | yes | 25 | rem | 82 | 1052 | 214% | VUS C3 | 2 | - | - |
|  | 230 | affected | F | spor | yes | 3 | rem | 57 |  | 177% | VUS C3 | 1 | - | - |
| #433 | 1080 | proband | F | spor | no | 1 | rem | 128 |  | 178% | no | 0 | 1 | 2 |
| #192 | 657 | proband | M | rec | no | 0.9 | rem | 92 | - | 201% | PV-CFI + PV MCP | 1 | 1 | - |
| #593 | 1299 | proband | F | rec | no | 34 | acute | 99 | 534 | 177% | VUS CFI | - | - | - |
| #736 | 1458 | proband | F | spor | no | 4 | rem | 82 |  | 249% | VUS CFH | - | - | - |

Bold: patients in remission, carrying PV in MCP only and risk haplotypes.

PV: pathogenetic variant; LPV: likely pathogenetic variant; VUS: variant of uncertain significance; LB: likely benign variant; B: benign variant.

Normal ranges: serum C3: 83-177 mg/dl; plasma sC5b-9: <400 ng/ml; C5b-9 deposition: <149%.

**Supplementary Table 2. Results of C5b-9 formation test in unaffected family members who do not carry the LPV or VUS identified in the proband of the same pedigree.**

| gene | nucleotide change | aminoacid change | classification | gnomAD frequency | CADD | C5b-9 formed  *(increase vs ctr=100)* | n° non-carriers positive C5b-9 test *(>149%)* |
| --- | --- | --- | --- | --- | --- | --- | --- |
| CFH | c.3544_3545insGGTGGACAGCCAAACAGAAGCTTTATTCGAGAACAG | p.R1182delinsRWTAKQKLYSRTG | LPV | - | - | 85% | 0/1 |
| CFH | c.2686_2700delAAATTGAGTTATACT | p.896_900delKLSYT | LPV | - | 16.80 | 79% | 0/2 |
|  |  |  |  |  |  | 91% |  |
| C3 | c.181G>A | p.D61N | LPV | 0.00001988 | 19.03 | 116% | 0/2 |
|  |  |  |  |  |  | 124% |  |
| CFH | c.127C>A | p.P43T | VUS | 0.000003983 | 20.9 | 105% | 0/1 |
| CFH | c.2171C>A | p.T724K | VUS | 0.00009201 | 24.1 | 113% | 0/1 |
| C3 | c.1898A>G | p.K633R | VUS | 0.0004565 | 0.043 | 99% | 0/1 |
| C3 | c.485C>G | p.T162R | VUS | - | 14.34 | 132% | 0/1 |
|  |  |  |  |  |  |  |  |
| Overall | | | | | | | 0/9 |

LPV: likely pathogenetic variant; VUS: variant of uncertain significant.

**Supplementary Figure 1**


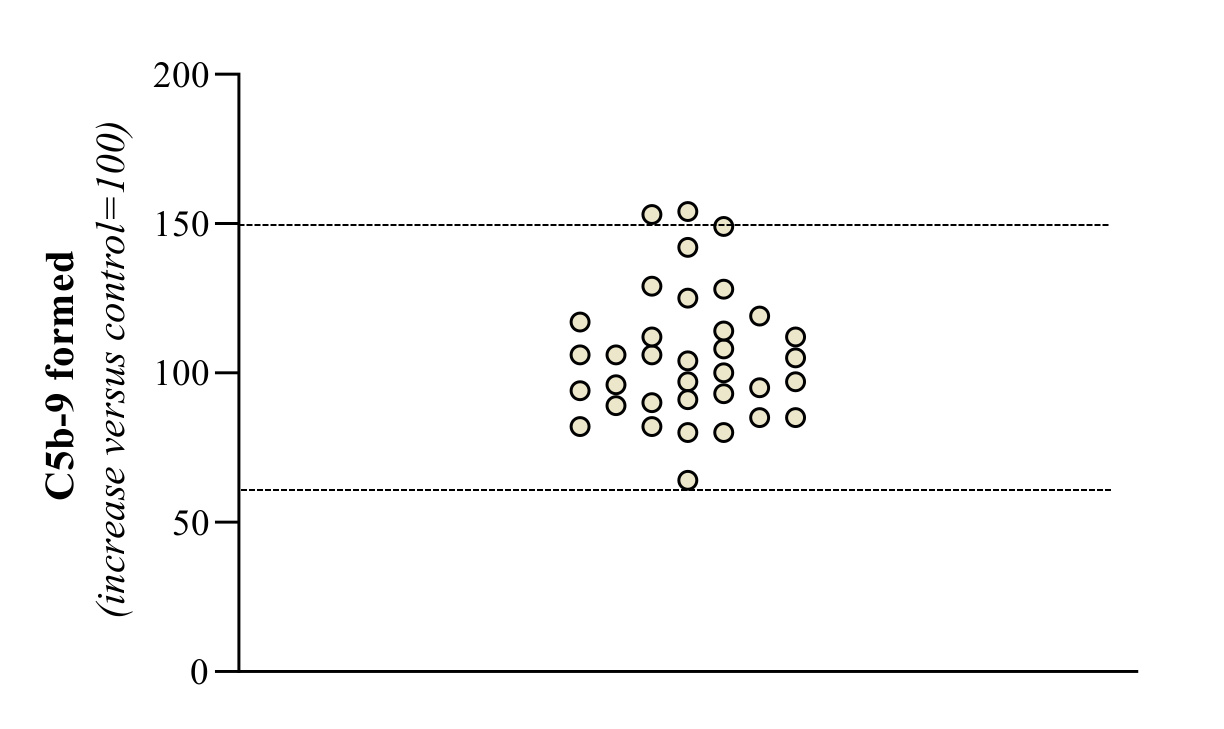


**C5b-9 formation on ADP-activated HMEC-1 exposed to control sera**

Endothelial surface area covered by C5b-9 staining after 2h incubation of ADP-activated HMEC-1 with serum (diluted 1:2 in test medium) from 35 healthy subjects. The results are shown as the fold increase of stained surface area after incubation with serum from single controls versus control pool of sera run in parallel. Points represent fold increase values of single subjects. Dotted lines are upper and lower limits of normal range.

**Supplementary figure 2**


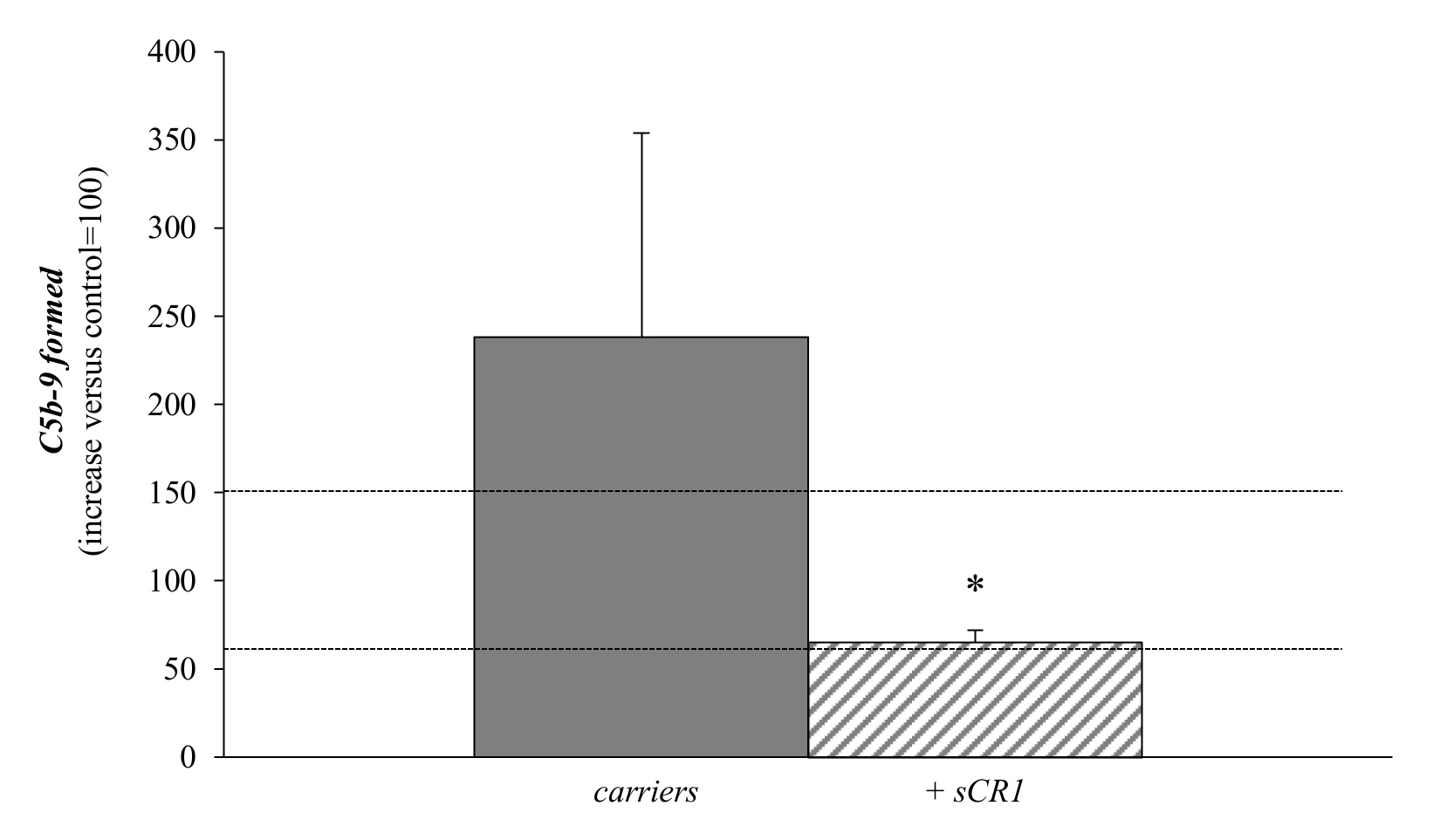


**Effect of sCR1 on C5b-9 formation on ADP-activated HMEC-1 exposed to serum from unaffected carriers of PV**

Endothelial surface area covered by C5b-9 staining after 2h incubation of ADP-activated HMEC-1 with serum (diluted 1:2 in test medium) from unaffected carriers of PV (n=6) in the presence or absence of the pan complement inhibitor sCR1. The results are shown as the fold increase of stained surface area after incubation with serum from unaffected carrier versus control pool of sera run in parallel. Data are mean±SD. Dotted lines are upper and lower limits of normal range. *P<0.01 versus carriers.

**Supplementary figure 3**


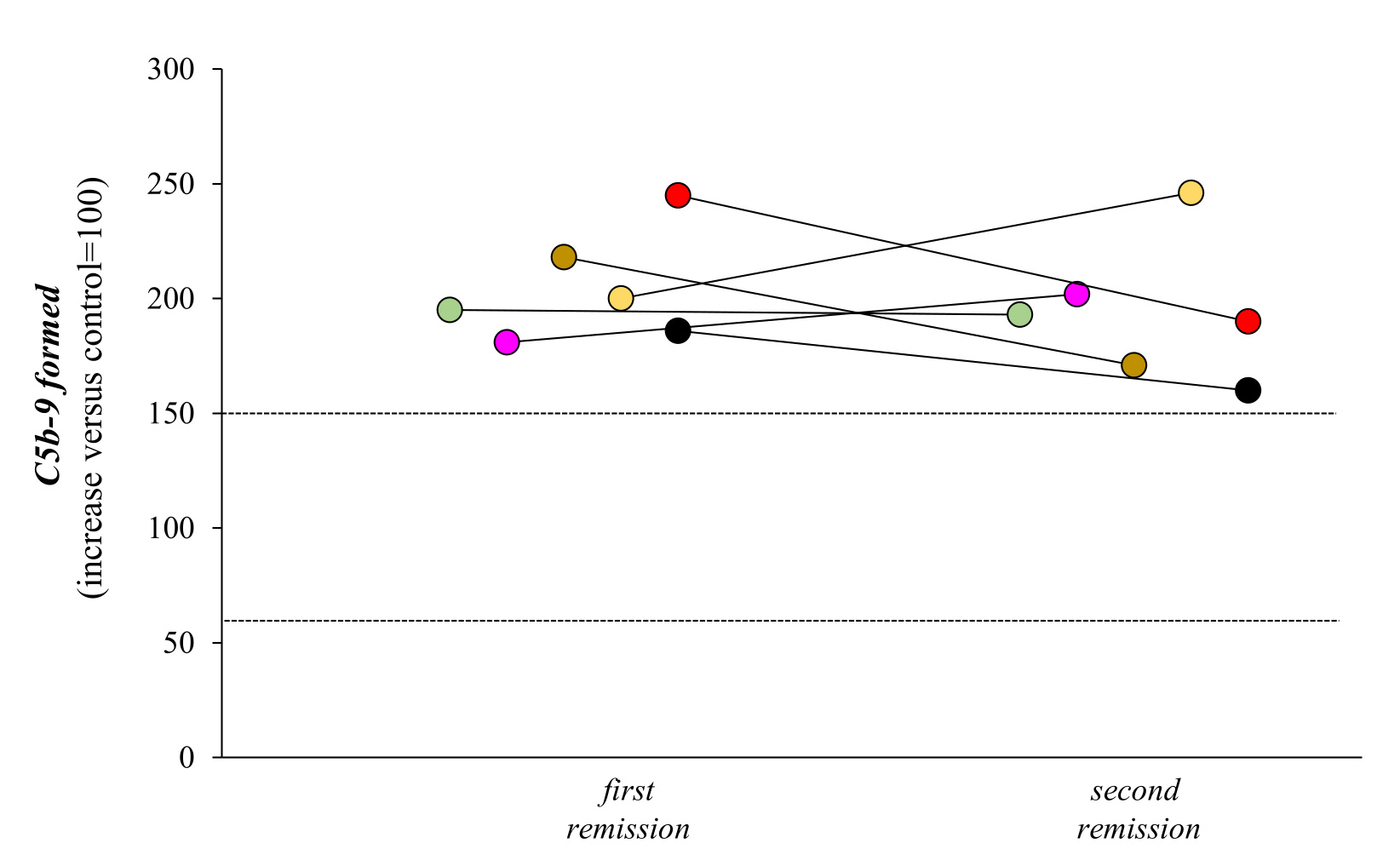


**C5b-9 formation on ADP-activated HMEC-1 exposed to serum from aHUS patients collected in remission at 2 different time points**

Endothelial surface area covered by C5b-9 staining after 2h incubation of ADP-activated HMEC-1 with serum (diluted 1:2 in test medium) from aHUS patients (n=6) collected at 2 different time points in remission. The results are shown as the fold increase of stained surface area after incubation with aHUS serum versus control pool of sera run in parallel. Points represent fold increase values of single subjects. Dotted lines are upper and lower limits of normal range.

**Supplementary figure 4**


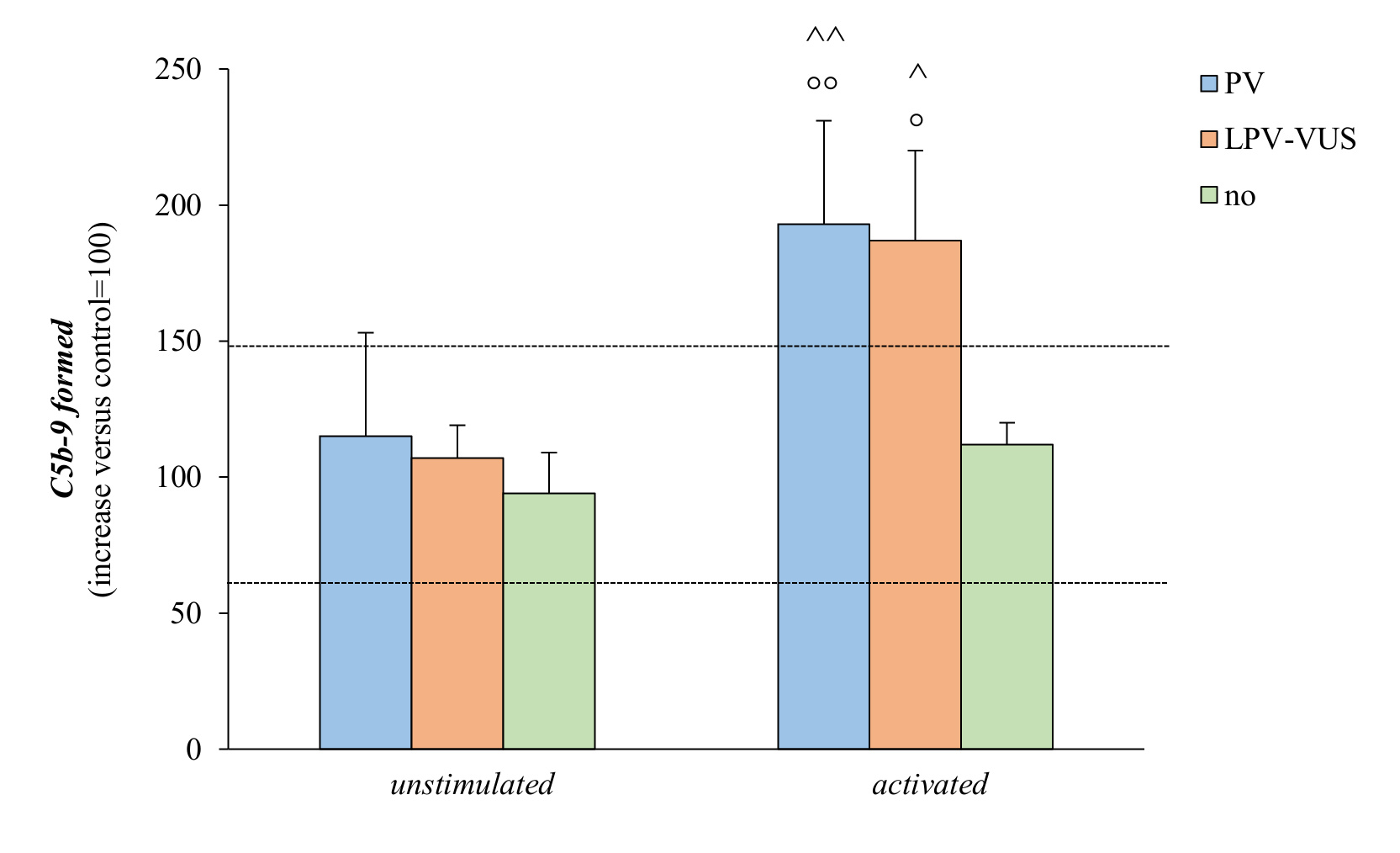


**C5b-9 formation on unstimulated or ADP-activated HMEC-1 exposed to serum from unaffected carriers**

Endothelial surface area covered by C5b-9 staining after 2h incubation of unstimulated or ADP-activated HMEC-1 with serum (diluted 1:2 in test medium) from unaffected carriers. The results are shown as the fold increase of stained surface area after incubation with serum from unaffected carrier versus control pool of sera run in parallel. Data are mean±SD (PV, n=3; LPV-VUS, n=7, no, n=7). Dotted lines are upper and lower limits of normal range. ^P<0.0001, ^^P<0.01 activated versus respective unstimulated; °P<0.0001, °°P<0.001 activated no variant. PV: pathogenetic variant; LPV: likely pathogenetic variant; VUS: variant of uncertain significance; no: no variant.
